# Semiconductive microporous hydrogen-bonded organophosphonic acid frameworks

**DOI:** 10.1038/s41467-020-16977-0

**Published:** 2020-06-23

**Authors:** Patrik Tholen, Craig A. Peeples, Raoul Schaper, Ceyda Bayraktar, Turan Selman Erkal, Mehmet Menaf Ayhan, Bünyemin Çoşut, Jens Beckmann, A. Ozgur Yazaydin, Michael Wark, Gabriel Hanna, Yunus Zorlu, Gündoğ Yücesan

**Affiliations:** 1 Technische Universität Berlin, Gustav-Meyer-Allee 25, 13355 Berlin, Germany; 2 University of Alberta, 116 St. and 85 Ave., Edmonton, AB T6G 2R3 Canada; 3 Carl von Ossietzky Universität Oldenburg, Carl-von-Ossietzky Str. 9-11, 26129 Oldenburg, Germany; 4 Gebze Technical University, Kimya Bölümü, 41400 Gebze-Kocaeli, Turkey; 5 University College London, Torrington Place, London, WC1E 7JE UK; 6 Universität Bremen, Leobener Str. 7, 28359 Bremen, Germany

**Keywords:** Chemistry, Materials science

## Abstract

Herein, we report a semiconductive, proton-conductive, microporous hydrogen-bonded organic framework (HOF) derived from phenylphosphonic acid and 5,10,15,20‐tetrakis[*p*‐phenylphosphonic acid] porphyrin (GTUB5). The structure of GTUB5 was characterized using single crystal X-ray diffraction. A narrow band gap of 1.56 eV was extracted from a UV-Vis spectrum of pure GTUB5 crystals, in excellent agreement with the 1.65 eV band gap obtained from DFT calculations. The same band gap was also measured for GTUB5 in DMSO. The proton conductivity of GTUB5 was measured to be 3.00 × 10^−6^ S cm^−1^ at 75 °C and 75% relative humidity. The surface area was estimated to be 422 m^2^ g^−1^ from grand canonical Monte Carlo simulations. XRD showed that GTUB5 is thermally stable under relative humidities of up to 90% at 90 °C. These findings pave the way for a new family of organic, microporous, and semiconducting materials with high surface areas and high thermal stabilities.

## Abstract (web summary)

Research in hydrogen-bonded organic frameworks (HOFs) has gained interest in recent years due to their facile design and synthesis but no semiconducting HOF has been reported to date. Here the authors report a thermally stable and proton-conductive organic semiconductor based on a porphyrin HOF.

## Introduction

Metal-organic frameworks (MOFs) emerged as revolutionary microporous materials at the beginning of the 21st century^1–3^. Owing to their well-ordered pores, which are surrounded by inorganic and organic components, MOFs have been used in a wide range of applications, such as gas storage/separation^4–7^, catalysis^8–13^, magnetism^14–16^, electric conductivity^17–19^, proton conductivity^20–22^, and drug delivery^23–25^. In parallel to MOF research, another closely related family of supramolecular architectures known as hydrogen-bonded organic frameworks (HOFs) has attracted immense interest in recent years^26–28^. In HOFs, the linker connectivity is achieved via hydrogen-bonded networks rather than inorganic building units (IBUs)^29–33^. Hydrogen bonds provide simpler connectivity options compared to the complex molecular, one-dimensional, two-dimensional, and three-dimensional IBUs of MOFs^34^. Therefore, the design and synthesis of stable hydrogen-bonded supramolecular networks can be more easily achieved compared to that of MOFs. HOFs are also more convenient to recycle and are free of heavy metal ions, providing environmentally friendly solutions. The recent interest in HOFs has resulted in several review articles^35–37^ summarizing their applications in CO_2_ capture^38–40^ and proton conductivity^41,42^. However, to date, no semiconducting HOFs have been reported in the literature. Thermally stable and permanently microporous semiconducting HOFs could revolutionize the design of supercapacitors and electrodes due to their simpler chemistry compared to MOFs. In this communication, we present the first example of a HOF (known as GTUB5, where TUB stands for Technische Universität Berlin and G for Gebze), synthesized using phosphonic acid functional groups (R-PO_3_H_2_), which exhibits a low band gap, proton conductivity, and high thermal stability.

The phosphonic acid functional group has two protons and one oxygen from the P = O bond, which allow it to form multiple hydrogen bonds with other phosphonic acid groups and thereby stabilize the resulting HOF. Interestingly, the unique structure and multiple metal-binding modes of the phosphonic acid functional group have led to some of the most thermally^34,43–46^ and chemically stable^34,47–49^ MOFs in the literature. The phosphonic acid functional group contains two deprotonation modes with p*K*_a_ values of 1.7 and 7.4, respectively^31^. Therefore, in order to synthesize our phosphonate HOF, we adopted a crystallization method at pHs between 1.7 and 7.4 with mixed phosphonic acid linkers of phenylphosphonic acid (PPA) and 5,10,15,20‐tetrakis [*p*‐phenylphosphonic acid] porphyrin (H_8_-TPPA) to ensure that at least one of the phosphonic acid moieties is not fully deprotonated. H_8_-TPPA exhibits a planar tetratopic geometry with a 90° angle between the phenylphosphonate tethers^49,50^. Given these starting conditions and materials, it is expected that a mixed linker strategy involving H_8_-TPPA and PPA could produce two-dimensional HOFs with hexagonal void channels.

## Results

### Design and structural characterization

**Fig. 1:**
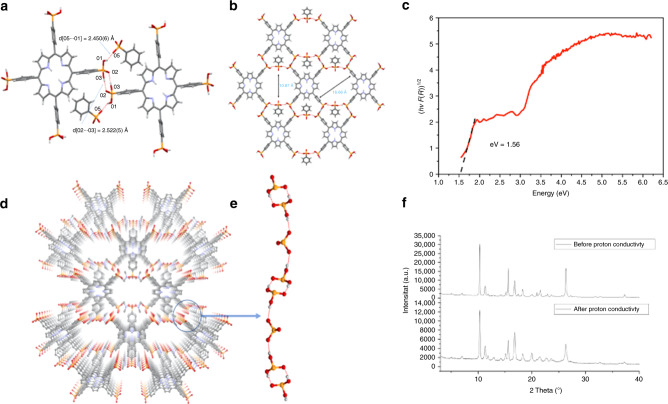
Structure of GTUB5, experimental band gap, and XRD patterns. **a** Portion of hydrogen-bonded network of GTUB5. **b** Depiction of hexagonal void spaces in GTUB5. **c** Tauc plot from the solid-state UV–Vis spectrum of GTUB5, showing a band gap of 1.56 eV. The second jump at 2.88 eV corresponds to the Soret band of the porphyrin core at 430 nm. **d** Layer structure of GTUB5. **e** One-dimensional hydrogen-bonded building unit of GTUB5. **f** XRD pattern before and after the proton conductivity measurements.

The H_8_-TPPA linker was synthesized according to our previously reported method involving a Pd-catalyzed Arbuzov reaction^50^, in order to avoid the porphyrin core being occupied by Ni(II) after a Ni-catalyzed Arbuzov reaction^49,50^. The synthesized metal-free H_8_TPPA linker eliminated the possibility of metal–ligand interactions that could have triggered the formation of MOFs. GTUB5 was synthesized following a conventional MOF crystallization method in scintillation vials in DMF/EtOH at a pH between 1.7 and 7.44 to ensure the presence of protonated phosphonic acid functional groups^32^. The synthesis of GTUB5 is quite facile, giving dark purple 1–2 mm long needle-shaped crystals in high yield (see “Methods” section for synthesis details). The dark purple color of GTUB5 is an indication of its semiconductive nature. The structure of GTUB5 was characterized using single crystal X-ray diffraction. As seen in Fig. 1a, b, GTUB5 is composed of two-dimensional sheets of hydrogen-bonded H_8_-TPPA and PPA moieties. The structure contains two different hydrogen-bonding patterns, which are observed between different H_8_-TPPA units and between H_8_-TPPA and PPA (see Fig. 1e). In the first pattern, the P = O bond from the H_8_-TPPA unit is exclusively involved in creating the (almost linear) double hydrogen-bonding pattern between each unit. In the second pattern, the hydrogen bond forms between the second protonated hydroxyl group of the H_8_-TPPA and the deprotonated PPA^2−^. The four DMF solvent molecules in the HOF structure act as a Lewis base acquiring the PPAs’ protons. The Brunauer–Emmett–Teller (BET) surface area of GTUB5 was estimated to be 422 m^2^ g^−1^ from a simulated N_2_ adsorption isotherm at 77 K (see Supplementary Fig. 3) obtained using the grand canonical Monte Carlo method (see “Methods” section for simulation details).

### Band gap measurements

The band gap was estimated from a solid-state diffuse reflectance UV–Vis spectrum of the GTUB5 crystals (see Supplementary Fig. 8). As seen in Fig. 1c, the Tauc plot derived from the spectrum yields a narrow band gap of 1.56 eV. The second jump at 2.88 eV corresponds to the Soret band of the porphyrin core at 430 nm. A similar band gap was also obtained from a UV–Vis spectrum of a dissolved sample of GTUB5 in DMSO (see Supplementary Fig. 7), suggesting that the hydrogen-bonded supramolecular structure is not disrupted in a polar aprotic solvent. From a cyclic voltammetry measurement on GTUB5 in DMSO (see Supplementary Fig. 9), the first oxidation and reduction potentials were measured to be 0.42 and −1.23 V, respectively, yielding a HOMO–LUMO gap of 1.65 eV, thereby supporting this hypothesis.

### Density functional theory (DFT) calculations

**Fig. 2:**
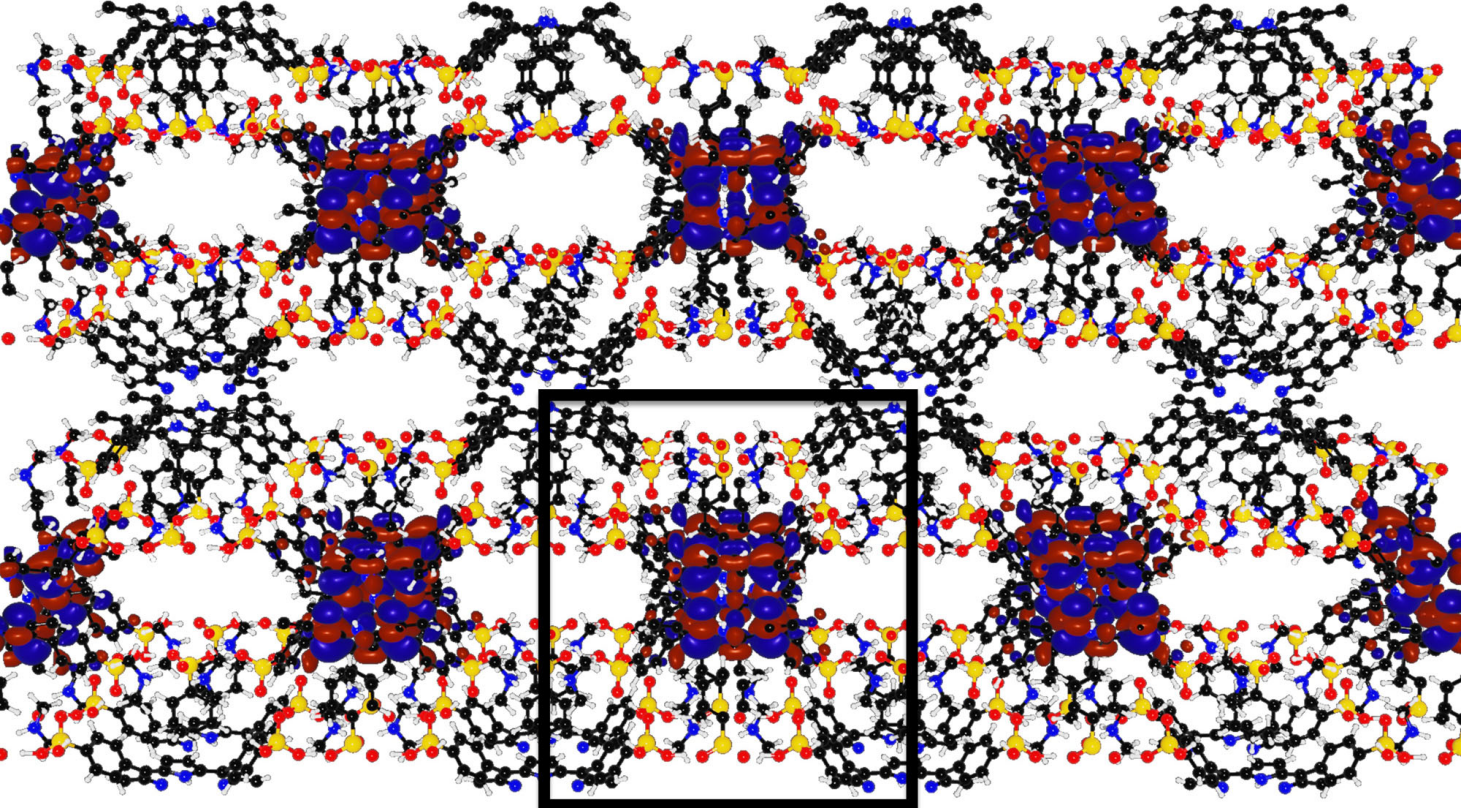
Periodic representation of GTUB5, with the unit cell delineated by the black box. The HOMO iso-surface corresponding to an electron density of 0.01 electrons per Å^3^ (negative and positive phases are shown in red and blue, respectively) is also shown (O—red; N—blue; P—yellow; C—black; H—white).

To gain insight into the semiconductive nature of GTUB5, we performed DFT calculations. The details of the calculations, employing hybrid Gaussian plane-wave (GPW) and Slater-type orbital (STO) basis sets, can be found in the “Methods” section. Figure 2 shows a periodic representation of the optimized geometry, which is in close agreement with the experimental crystal structure (see Supplementary Table 3 and Supplementary Figs. 4–7). A single point calculation on the optimized structure yields a band gap of 1.65 eV, in very good agreement with the experimental result of 1.56 eV. As seen in Fig. 2, the HOMO and LUMO are predominantly localized on some of the porphyrins within the supercell (in which, a single unit cell is delineated by the black rectangle), but not all of them; with the LUMO localized on the same porphyrins as the HOMO. The fact that the HOMO and LUMO are aligned along the axis perpendicular to the plane of the page within the supercell suggests that GTUB5 is only conductive along this direction.

**Fig. 3:**
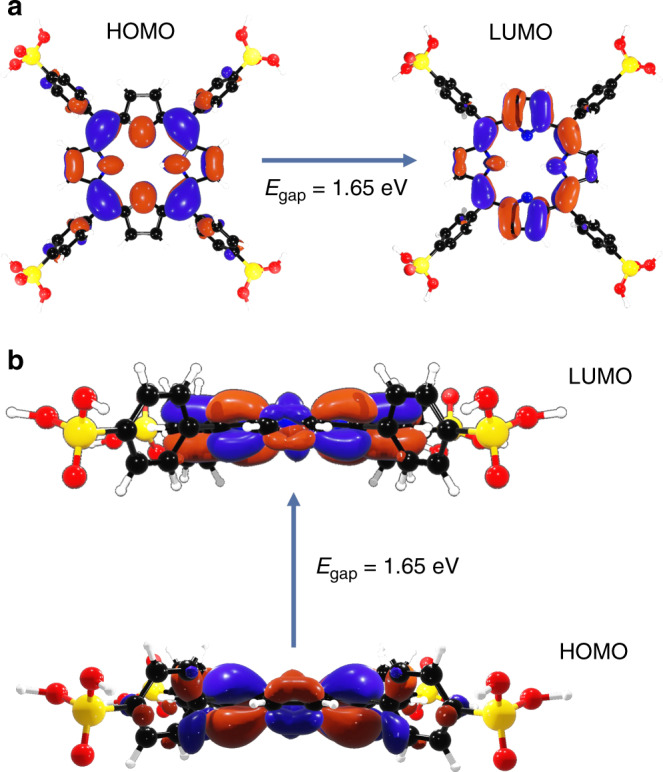
HOMO and LUMO iso-surfaces, corresponding to an electron density of 0.01 electrons per Å^3^. **a** Top view. **b** Side view. Red/blue correspond to the negative/positive phases (O—red; N—blue; P—yellow; C—black; H—white) .

**Fig. 4:**
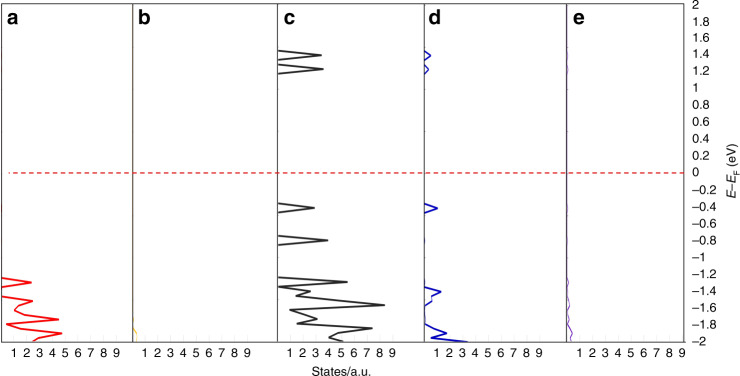
Atom-specific projected density of states (pDOS) for GTUB5, generated using ADF-BAND51. Projected density of states (pDOS) for **a** O, **b** P, **c** C, **d** N, and **e** H in GTUB5, generated using ADF-BAND^51^.

Focusing in on the portions of the structure that have significant HOMO and LUMO density, we see that the HOMO and LUMO are indeed localized on the same porphyrin (see Fig. 3). Moreover, they are mostly confined to a subset of the carbons and nitrogens. The HOMO is composed of π orbitals mostly on *sp*^2^ hybridized carbons and nitrogens, while the LUMO is composed of π* orbitals on some of the *sp*^2^ carbons and nitrogens. As shown in Table 1, ∼75% of the HOMO and LUMO orbital contributions are from the carbon and nitrogen 2*p* orbitals of the porphyrin. Table 1 also shows that a HOMO–LUMO transition would lead to an increase in the carbon 2*p*_*x*_ orbital population, a slight decrease in the carbon 2*p*_*y*_ population, and a slight increase in the carbon 2*p*_*z*_ population; while the nitrogen 2*p*_*x*_ and 2*p*_*z*_ populations both decrease (the 2*p*_*y*_ population remains negligible). These results suggest that the semiconductive nature of GTUB5 is predominantly determined by π–π* transitions involving orbitals localized on some of the porphyrin carbons and nitrogens. Inspection of the projected density of states (pDOS) confirms that the HOMO–LUMO gap is predominantly due to orbitals localized on carbons and nitrogens (see Fig. 4).

**Table 1:** Contributions from the 2*p* orbitals on the porphyrin carbons and nitrogens to the HOMO and LUMO.

	2*p*_*x*_	2*p*_*y*_	2*p*_*z*_	Sum
*Carbon*				
HOMO	0.366	0.042	0.134	0.541
LUMO	0.484	0.020	0.170	0.674
*Nitrogen*				
HOMO	0.163	4.70 × 10^−7^	0.053	0.216
LUMO	0.048	5.22 × 10^−4^	0.020	0.067

### Thermogravimetric analysis

The thermal decomposition of GTUB5 was investigated via a thermogravimetric analysis (TGA) of hand-picked crystals up to 900 °C. The TGA curve (see Supplementary Fig. 10) shows an initial 2% loss between 50 and 100 °C, corresponding to the evaporation of the remaining MeOH on the crystal surface after the synthesis. The following 12% step until 250 °C corresponds to the loss of dimethylammonium cations (calculated to be 12.9% based on the molecular formula). The remaining organic components of GTUB5 decompose in two steps at ca. 400 and 900 °C. The second large weight loss at ca. 900 °C suggests the formation of phosphides above 400 °C^52^.

### Proton conductivity

Given the presence of –PO_3_H_2_ groups in its hydrogen-bonded framework, the proton conductivity of GTUB5 was measured. Electrochemical impedance spectroscopy measurements were carried out at 75% and 90% relative humidity (%rh) and temperatures in the range of 25–75 °C (see Supplementary Information and ref. ^52^ for setup details). At 75%rh, we see that the proton conductivity of GTUB5 increases from 8.29 × 10^−7^ to 3.00 × 10^−6^ S cm^−1^ as the temperature is increased from 25 to 75 °C, while at 90%rh the conductivities are higher but the increase is more moderate (see Table 2 for full data set). The activation energies, i.e., sum of the migration and defect formation energies, were extracted from the slopes of the Arrhenius plots (see Supplementary Fig. 15) to be *E*_A_ = 0.26 eV and *E*_A_ = 0.14 eV at 75 and 90 °C, respectively. These low activation energies suggest that a Grotthuss mechanism with high proton mobility (and therefore low migration energy) and H-bridges to water molecules is the predominant mechanism for proton conduction through the framework. As seen in Fig. 1f, the XRD pattern of the sample recorded before and after the proton conductivity experiments slightly changes, indicating that the structure was slightly affected by the humidified atmosphere and the applied temperatures up to 75 °C during the measurements.

**Table 2:** Proton conductivities and activation energies (*E*_A_) of GTUB5 at different relative humidities.

Relative humidity [%rh]		75	90
Conductivity [S cm^−1^]	25 °C	8.29 × 10^−7^	3.55 × 10^−6^
	50 °C	1.67 × 10^−6^	3.26 × 10^−6^
	75 °C	3.00 × 10^−6^	4.20 × 10^−6^
*E*_A_ [eV]		0.26	0.14

Due to the large numbers of phosphonate groups and hydrogen bonds in GTUB5, one might expect higher proton conductivities. However, in addition to the numbers of phosphonate groups and hydrogen bonds, one must also (and more importantly) consider how rigidly the phosphonate groups are connected to the framework, i.e., how much they change their positions (through rotations and vibrations), and, in turn, how strongly they interact with the water molecules. In a previous study, we found comparable proton conductivities (viz., 1.35 × 10^−6^ S cm^−1^ at 75% rh and 80 °C and 5.62 × 10^−6^ S cm^−1^ at 90% rh and 80 °C) for porous porphyrin-based metal tetraphosphonates of the CAU-29 type whose phosphonates sit similarly close to the network^53^. If, however, proton-conducting groups are linked to the framework via flexible vibrating and rotating alkyl chains, like in SO_3_H-propylsilane modified Si-MCM-41, proton conductivities higher than 10^−3^ S cm^−1^ at 98% rh and 80 °C could be achieved^54^. As quantum chemical calculations in that study confirmed, the proton conductivity is not only strongly dependent on the density of the proton-conducting groups per nm^2^, but also on the length of the alkyl chain connecting the proton-conducting group with the rigid framework. In the cases of GTUB5 and CAU-29, no such flexible alkyl chains are present.

## Discussion

Herein, we presented GTUB5, a two-dimensional, microporous phosphonic acid HOF (with a calculated surface area of 422 m^2^ g^−1^). Given its low band gap (as confirmed by solid-state/solution measurements and DFT calculations), GTUB5 paves the way for the creation of new semiconductive microporous organic compounds. The use of hydrogen bonds in constructing a framework comes with the advantages of simpler connectivity options and no toxic metal ions (which could possibly lead to environmentally friendly solutions for capacitors and batteries). The hydrogen-bonded framework also renders GTUB5 proton-conductive (with proton conductivities on the order of 10^−6^ S cm^−1^). Owing to its structure, the phosphonic acid group can give rise to structurally diverse frameworks, which could increase the number of potential HOF applications. GTUB5 has the same band gap of 1.65 eV in DMSO, suggesting that GTUB5 retains its hydrogen-bonded network in polar aprotic solvents. Therefore, phosphonate HOFs have the potential to revolutionize the semiconductive materials industry with applications in printed electronics, optoelectronics, photovoltaics, and electrodes in supercapacitors. Currently, we are focusing on different linker geometries and pH modulations to further optimize the pore sizes and conductive behavior of phosphonate HOFs.

## Methods

### Synthesis

All the reagents and solvents employed were commercially available and used as received without further purification. The linker H_8_TPPA was synthesized according to our previously reported method^50^ (8.77 mg, 0.0088 mmol) and phenylphosphonic acid (PPA) (208 mg, 1.3 mmol) in a 1.6 mL mixture of DMF/EtOH or DMF/MeOH (1.36:0.24, v/v) were added to a 5-mL glass vial. The reaction mixture was ultrasonically dissolved and then heated to 80 °C in an oven for 48 h. After cooling down to room temperature, dark purple block crystals of GTUB5 formed, which were then isolated by filtration, washed with DMF and acetone, and finally air-dried. The yield of GTUB5 was 5 mg.

### Single crystal structure solution

Suitable single crystals of GTUB5 with appropriate dimensions (0.43 mm × 0.14 mm × 0.12 mm) were carefully chosen from the glass vial using a polarizing microscope, coated with perfluoropolyether oil in order to eliminate the possibility of decomposition, and finally mounted to a thin glass fiber. Intensity data collection was performed with a Bruker APEX II QUAZAR three-circle diffractometer equipped with the IμS Incoatec Microfocus Source with Mo-Kα radiation (*λ* = 0.710723 Å) at room temperature (296 K). Indexing was performed using APEX2^55^. Data integration and reduction were carried out with SAINT^56^. Absorption correction was performed by multi-scan method implemented in SADABS^57^. The structure was solved using SHELXT^58^ and then refined by full-matrix least-squares refinements on F^2^ using SHELXL^59^ in the Olex2 Software Package^60^. The positions of all H-atoms bonded to the carbon, nitrogen, and oxygen atoms were geometrically optimized with the following HFIX instructions in SHELXL: HFIX 23 for the –CH_2_– moieties, HFIX 137 for the –CH_3_, HFIX 43 for the CH and NH groups of the aromatic rings and porphyrin cores, and HFIX 147 for the –P–OH groups (H1a) of the phosphonic acid moieties. Another O-bound H atom (H3) was located from the difference Fourier-map. Finally, their displacement parameters were set to isotropic thermal displacements parameters [*U*_iso_(H) = 1.2*U*_eq_ for CH, NH, and CH_2_ groups and *U*_iso_(H) = 1.5*U*_eq_ for OH and CH_3_ groups]. In the chemical formula [(H_8_-TPPA)(PPA)_2_(DMA)_4_] of GTUB5, the H_8_-TPPA building block is not deprotonated, while the protons of the phenylphosphonic acid (PPA) groups are acquired by the DMF solvent in the pores to form four dimethylammonium cations (DMA–[NH_2_(CH_3_)_2_]^+^) to balance the charge. SQUEEZE was used to remove the electron density caused by seriously disordered solvent molecules in GTUB5. Along the *c*-axis, the 3D supramolecular network of GTUB5 produced a one-dimensional distinctive void space with a total potential solvent area occupying 19.2% (785 Å^3^) of the unit cell volume (4081.7 Å^3^), obtained using the PLATON software package^61^. Analysis of solvent accessible voids in the structure was performed using CALC SOLV in PLATON with a probe radius of 1.20 Å and grid spacing of 0.2 Å. Van der Waals (or ion) radii used in the analysis are 1.70 Å for C, 1.20 Å for H, 1.55 Å for N, 1.52 Å for O, and 1.80 Å for P. Also, in this crystal structure, the rotationally disordered phosphonate part (–PO_3_) in PPA was refined as 0.77:0.23. Crystallographic data and refinement details of the data collection for GTUB5 are given in Supplementary Table 4. Crystal structure validations and geometrical calculations were performed using PLATON^61^. The Mercury software package^62^ was used for visualization of the cif files.

### Grand canonical Monte Carlo (GCMC) surface area calculations

The surface area of GTUB5 was calculated by force-field based atomistic simulations, which were performed with the RASPA molecular simulation package^63^. For these simulations, the GTUB5 unit cell was replicated by 1 × 2 × 4 times in the *x*, *y*, and *z* directions, respectively, and the replicated framework atoms were fixed in their crystallographically determined positions. Lennard–Jones (LJ) and Coulomb potentials were employed to determine the non-bonded interaction energies between atoms:

1 $$V_{ij} = 4\varepsilon _{ij}\left[ {\left( {\frac{{\sigma _{ij}}}{{r_{ij}}}} \right)^{12} - \left( {\frac{{\sigma _{ij}}}{{r_{ij}}}} \right)^6} \right] + \frac{{q_i\,q_j}}{{4\,\varepsilon _0\,r_{ij}}}$$

where *r*_*ij*_ is the distance between atoms *i* and *j*, *ε*_*ij*_ and *σ*_*ij*_ are the LJ well depth and diameter, respectively, *q*_*i*_ is the partial charge of atom *i*, and *ε*_0_ is the dielectric constant. The LJ parameters between different types of sites were calculated using the Lorentz–Berthelot mixing rules, and the Ewald summation method was employed to compute the electrostatic interactions. The LJ interactions were shifted to be 0 at a cutoff distance of 12.0 Å. For the real part of the Ewald summation, the cutoff was also set to 12.0 Å.

A N_2_ adsorption isotherm for GTUB5 was computed by performing GCMC simulations at 77 K and up to 0.4 bar. In the GC ensemble, the chemical potential, volume, and temperature of the system are fixed; however, the number of molecules fluctuates. For all GCMC simulations, a 100,000 cycle initialization and a 100,000 cycle production run were performed. Each cycle is *N* steps, where *N* is equal to the number of molecules in the system. Random insertions, deletions, translations, rotations, and reinsertions of the N_2_ molecules were sampled with equal probability. The TraPPE force field was used to model the N_2_ molecules^64^, which was originally fit to reproduce the vapor–liquid coexistence curve of N_2_. In this force field, the N_2_ molecule is rigid with the N–N bond length fixed at its experimental value of 1.10 Å. This model reproduces the experimental gas-phase quadrupole moment of the N_2_ molecule by placing partial charges on nitrogen atoms and on a point located at the center of mass (COM) of the molecule. Supplementary Table 2 shows the LJ parameters and partial charges for the N_2_ molecule. In GCMC simulations, one computes the absolute adsorption (*N*_total_); whereas, in adsorption experiments, the excess adsorption (*N*_excess_) is measured. Therefore, the simulated excess adsorption of N_2_ was calculated using the following expression:

2 $$N_{{\mathrm{total}}} = N_{{\mathrm{excess}}} + \,\rho _{{\mathrm{gas}}}V_{\mathrm{{{p}}}}$$

where *ρ*_gas_ is the bulk density of the gas at simulation conditions which were calculated using the Peng–Robinson equation of state and *V*_p_ is the accessible pore volume. The BET surface area of GTUB5 was obtained from the simulated N_2_ adsorption isotherm (see Supplementary Fig. 3) and estimated to be 422 m^2^ g^−1^. When applying the BET theory, we made sure that our analysis satisfied the two consistency criteria as detailed by Walton and Snurr^65^.

### Density functional theory (DFT) calculations

The geometry optimization of GTUB5 was performed using DFT and the conjugate gradient method^66^ within the Quickstep-CP2K program^67,68^, starting from the experimental crystal structure and with the lattice vectors set to their experimental values. Since GTUB5 is a bulk material, periodic boundary conditions were applied to a reoriented 1 × 1 × 1 cell (*a* = 25.452 Å, *b* = 22.863 Å, *c* = 7.1798 Å, *α* = *γ* = 90.0°, *β* = 102.325°). The Perdew–Burke–Ernzerhof (PBE)^69^ generalized gradient approximation (GGA) functional was used in conjunction with the Grimme D3 dispersion correction^70^ and BJ damping^71^. The Gaussian and plane waves method^70,72^ was used, with the valence orbitals expanded in terms of molecularly optimized Gaussian basis sets of double-ζ plus polarization (MOLOPT-DZVP)^73^ quality and the core electrons represented by norm-conserving Goedecker–Teter–Hutter pseudopotentials^74,75^. Γ-point sampling was used and the plane-wave cutoff in reciprocal space was set to 550 Ry, with a Gaussian mapping of 60 Ry over five multi-grids. The self-consistent field was converged to 10^−6^ Ry with the FULL_ALL preconditioner using the orbital transformation method with a HOMO–LUMO gap of 1.67 eV. Single point calculations were performed using CP2K to obtain the HOMO–LUMO iso-surface plots (Figs. 2 and 3), orbital populations (Table 1), and the HOMO–LUMO gap. Another single point calculation was performed using the Slater-Type Orbital (STO) software ADF-BAND 2018.104^76,77^ to obtain the projected density of states (pDOS) (Fig. 4), band structure (Supplementary Fig. 8), and the HOMO–LUMO gap. The periodic ADF-BAND calculations were performed using an all-electron double-ζ plus polarization (DZP) basis set, PBE-D3-BJ, and Γ-point sampling for the 1 × 1 × 1 unit cell, with a good numerical quality. The HOMO–LUMO gaps obtained from CP2K and ADF-BAND were both 1.65 eV (thus, the HOMO–LUMO iso-surfaces and orbital populations obtained from CP2K are expected to be the same as those from ADF-BAND).

## Supplementary information

Supplementary Information

## Data Availability

All data is available in the main text and the Supplementary Information. The source data for Table 1, Fig. 1c, Supplementary Figs. 2–4, 10, 13, 16, 17, and the CIF file are provided as a Source Data file. The X-ray crystallographic coordinates for the structure reported in this study can be obtained free of charge from the Cambridge Crystallographic Data Centre (CCDC) [under the deposition number CCDC: 1963794 for GTUB5] via www.ccdc.cam.ac.uk/data_request/cif.
