# Supplementary material for: Semiconductive microporous hydrogen-bonded organophosphonic acid frameworks
Source: Nat Commun. 2020 Jun 23;11:3180. doi: 10.1038/s41467-020-16977-0 (PMC7311548; doi:10.1038/s41467-020-16977-0)
Supplement: Supplementary file 1 — Supplementary Information [file 41467_2020_16977_MOESM1_ESM.pdf]

Supplementary Information for

Semiconductive Microporous Hydrogen-Bonded Organophosphonic Acid Frameworks

Patrik Tholen[1], Craig A. Peeples[2], Raoul Schaper[3], Ceyda Bayraktar[4], Turan Selman Erkal[5], Menaf Ayhan[4], Bünyemin Çoşut[4], Jens Beckmann[6], A. Ozgur Yazaydin[5], Michael Wark[3], Gabriel Hanna[2], Yunus Zorlu[4]*, Gündoğ Yücesan[1]*

Affiliations:

[1]Technische Universität Berlin, Gustav-Meyer-Allee 25, 13355 Berlin, Germany

[2]University of Alberta, 116 St. and 85 Ave. Edmonton, Alberta T6G 2R3, Canada

[3]Carl von Ossietzky Universität Oldenburg, Carl-von-Ossietzky Str. 9-11, 26129 Oldenburg, Germany

[4]Gebze Technical University, Kimya Bölümü, 41400 Gebze-Kocaeli, Turkey

[5]University College London, Torrington Place, London WC1E 7JE, United Kingdom

[6]Universität Bremen, Leobener Str. 7, 28359 Bremen, Germany

*Correspondence to: yuecesan@tu-berlin.de and yzorlu@gtu.edu.tr

This PDF file includes:

Supplementary Methods

Supplementary Figures 1 to 17

Supplementary Tables 1 to 5

Supplementary Methods

Synthesis of GTUB5

All the reagents and solvents employed were commercially available and used as received without further purification. As can be seen in Supplementary Figure 1, 5,10,15,20-Tetra(p-bromophenyl)porphyrin (**TBPP**) and phosphonate-functionalized porphyrins (**TDPP**, **TPPP**, **H_8-TPPA**) were synthesized employing our research methodologies[1]. To synthesize **GTUB-5**, **H_8-TPPA** (8.77 mg, 0.0088 mmol) and phenylphosphonic acid (PPA) (208 mg, 1.3 mmol) in a 1.6 mL mixture of DMF/EtOH or DMF/MeOH (1.36:0.24, v/v) were added to a 5-mL glass vial. The reaction mixture was ultrasonically dissolved and then heated to 80 °C in an oven for 48 h. After cooling down to room temperature, dark purple block crystals of GTUB5 formed, which were then isolated by filtration, washed with DMF and acetone, and finally air-dried. The yield of GTUB5 was ~5 mg.

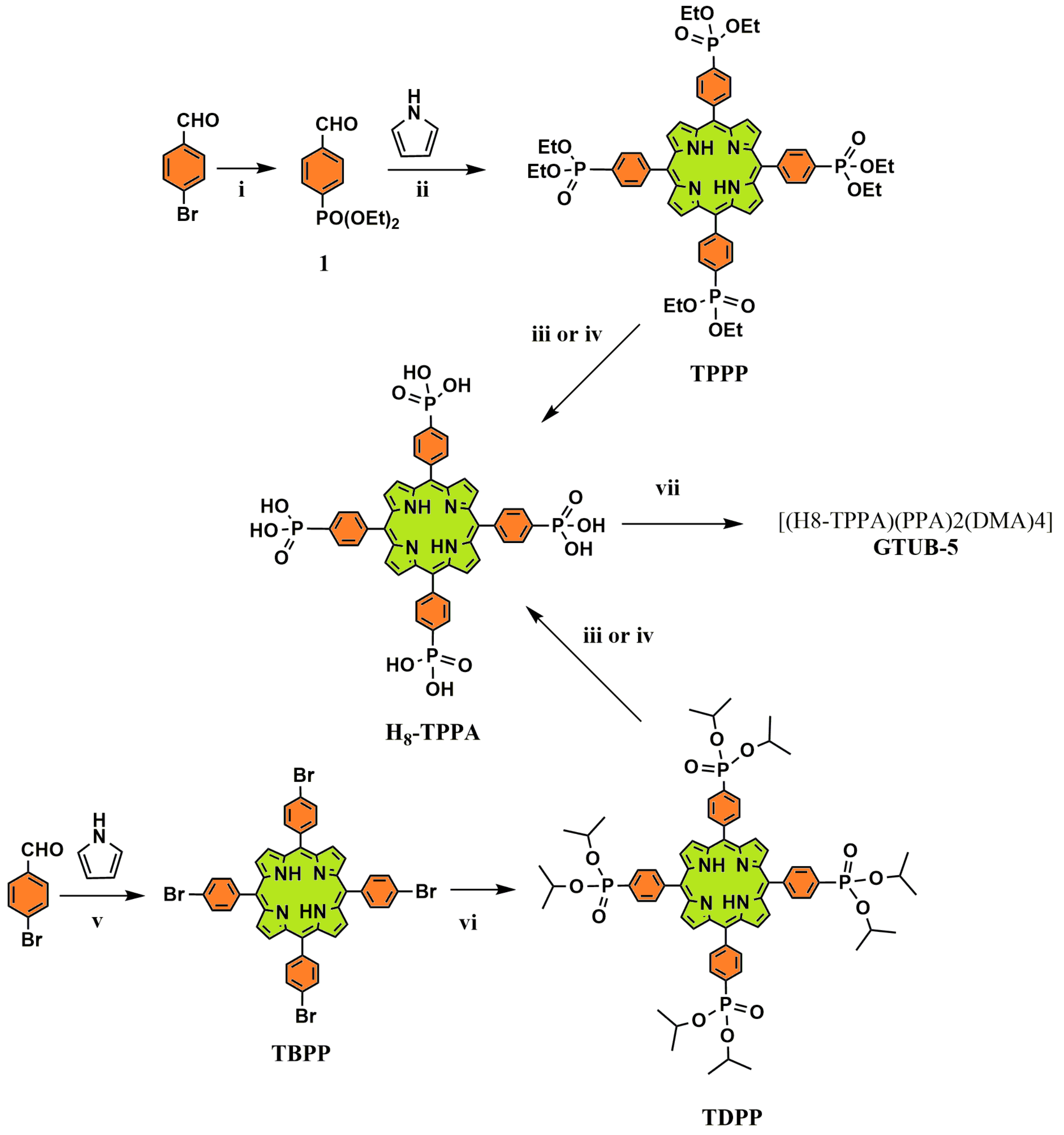

Supplementary Figure 1. Synthetic route for meso-aryl phosphonate-functionalized porphyrin derivatives and the porous phosphonic acid hydrogen-bonded organic framework **GTUB-5**.

Molecular simulations

The accessible pore volume, pore size distribution, and surface area of **GTUB-5** were calculated by force-field based atomistic simulations, which were performed with the RASPA molecular simulation package[2]. For these simulations, the **GTUB-5** unit cell was replicated by 1 x 2 x 4 times in the *x*, *y*, and *z* directions, respectively, and the replicated framework atoms were fixed in their crystallographically determined positions. Lennard-Jones (LJ) and Coulomb potentials were employed to determine the non-bonded interaction energies between atoms:

$$V_{ij} = 4\varepsilon_{ij}\left[\left(\frac{\sigma_{ij}}{r_{ij}}\right)^{12} - \left(\frac{\sigma_{ij}}{r_{ij}}\right)^{6}\right] + \frac{q_i\, q_j}{4\,\varepsilon_0\, r_{ij}} \qquad (1)$$

where r_{ij} is the distance between atoms i and j, ε_{ij} and σ_{ij} are the LJ well depth and diameter, respectively, q_i is the partial charge of atom i, and ε_0 is the dielectric constant. In all simulations, the LJ parameters between different types of sites were calculated using the Lorentz-Berthelot mixing rules, and the Ewald summation method was employed to compute the electrostatic interactions. The LJ interactions were shifted to be 0 at a cutoff distance of 12.0 Å. For the real part of the Ewald summation, the cutoff was also set to 12.0 Å.

LJ parameters for the **GTUB-5** atoms (See Supplementary Table 1) were taken from the DREIDING[3] force field. Partial atomic charges for the framework atoms were obtained with the REPEAT method[4], which fits point charges against the electrostatic potential. The electrostatic potential of **GTUB-5** was obtained from a single-point energy calculation using periodic plane-wave DFT with the CASTEP 17.21 software[5] and by employing the PBE[6] functional and ultrasoft pseudopotentials[7] with a 550 eV cutoff.

Supplementary Table 1. LJ parameters for the framework atoms of **GTUB-5**

Atom type	σ (Å)	$\boldsymbol{\varepsilon/k_B}$ (K)
C	3.473	47.856
O	3.033	48.158
H	2.846	7.649
P	3.695	153.476

Accessible pore volume. The accessible pore volume of **GTUB-5** was computed with the Widom insertion method using a helium probe[8], and estimated to be 0.176 cm^3 g^{-1}. This calculation involved averaging over 100,000 random insertions of a single helium atom into the framework. Then, the specific pore volume, i.e., pore volume available per unit mass, was determined by

$$V_p = \frac{1}{m_s} \int e^{-\varphi(r)/kT} \, dr \qquad (2)$$

where ϕ is the helium-solid interaction potential for a single helium atom, dr is a differential volume element, and m_s is the mass of the solid adsorbent in the simulation box. The LJ parameters for helium were taken from Hirschfelder et al. [9], and are σ_{He}= 2.640 Å and ε_{He}/k_B= 10.9 K.

Pore size distribution. The pore size distribution of **GTUB-5** (See Supplementary Figure 2) was computed with the method of Gelb and Gubbins[10]. Briefly, this method considers subvolumes of the system accessible to spheres of different radii. Let $V_{pore}(r)$ be the volume of the void space "accessible" by spheres of radius r or smaller; a point x can only be considered in $V_{pore}(r)$ if we can construct a sphere of radius r that overlaps x and does not overlap any framework atoms. The derivative $-dV_{pore}(r)/dr$ is the fraction of volume accessible by spheres of radius r but not by spheres of radius $r + dr$ and is a direct

definition of the pore size distribution. $V_{pore}(r)$ was calculated by Monte Carlo volume integration (10,000 iterations) and setting dr=0.12 Å.

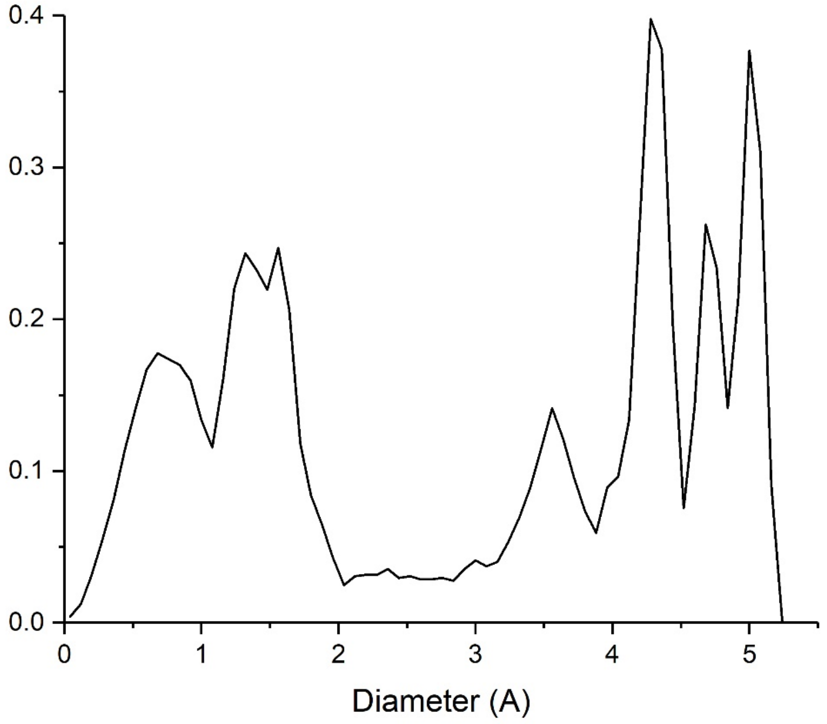

Supplementary Figure 2. Pore size distribution of **GTUB-5**.

N_2 adsorption isotherm and BET surface area. A simulated N_2 adsorption isotherm of **GTUB-5** was computed by performing grand canonical Monte Carlo (GCMC) simulations at 77 K and up to 0.4 bar. In the GC ensemble, the chemical potential, volume, and temperature of the system are fixed; however, the number of molecules fluctuates. For all GCMC simulations, a 100,000 cycle initialization and a 100,000 cycle production run were performed. Each cycle is N steps, where N is equal to the number of molecules in the system. Random insertions, deletions, translations, rotations, and reinsertions of the N_2 molecules were sampled with equal probability. The TraPPE force field was used to model the N_2 molecules[11], which was originally fit to reproduce the vapor-liquid coexistence curve of N_2. In this force field, the N_2 molecule is rigid with the N-N bond length fixed at its experimental value of 1.10 Å. This model reproduces the experimental gas-phase quadrupole moment of the N_2 molecule by placing partial

charges on nitrogen atoms and on a point located at the center of mass (COM) of the molecule. Supplementary Table 2 shows the LJ parameters and partial charges for the N_2 molecule.

Supplementary Table 2. LJ parameters and partial charges for the sites in the N_2 molecule

	σ (Å)	$\boldsymbol{\varepsilon/k_\mathrm{B}}$ (K)	q (e)
N	3.31	36.0	-0.482
N_2 COM	0	0	0.964

Using GCMC simulations, one can compute the absolute adsorption (N_{total}); whereas, in adsorption experiments, the excess adsorption (N_{excess}) is measured. Therefore, the simulated excess adsorption of N_2 was calculated using the following expression

$$N_{\mathrm{total}} = N_{\mathrm{excess}} + \rho_{\mathrm{gas}} V_{\rho} \quad (3)$$

where ρ_{gas} is the bulk density of the gas at simulation conditions which were calculated using the Peng-Robinson equation of state and V_{p} is the accessible pore volume. The BET surface area of **GTUB-5** was obtained from the simulated N_2 adsorption isotherm of **GTUB-5** (See Supplementary Figure 3) and estimated to be 422 m^2 g^{-1}. When applying the BET theory, we made sure that our analysis satisfied the two consistency criteria as detailed by Walton[12] et al.

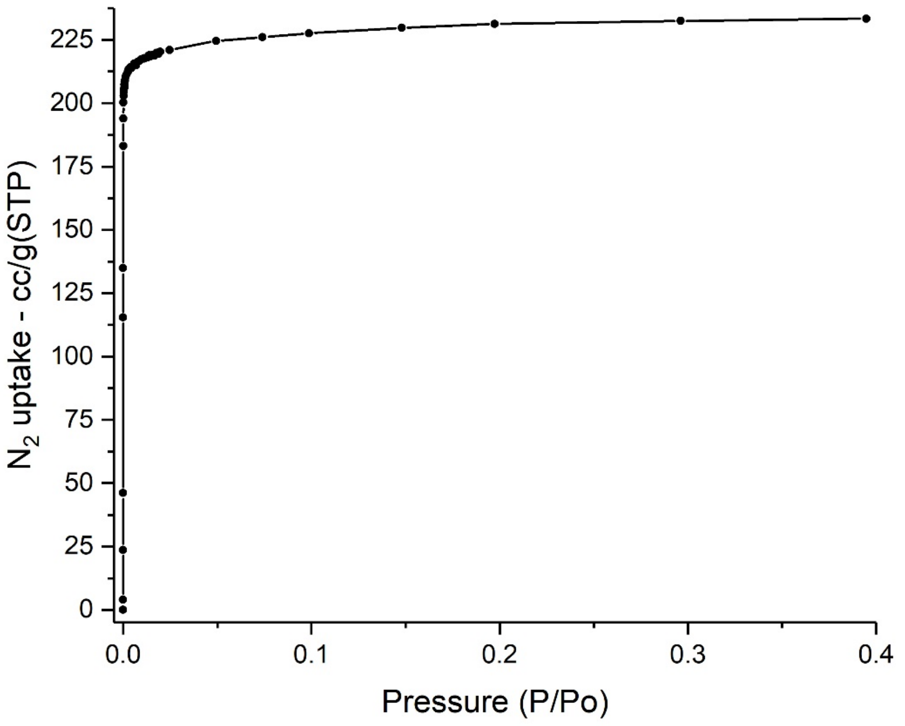

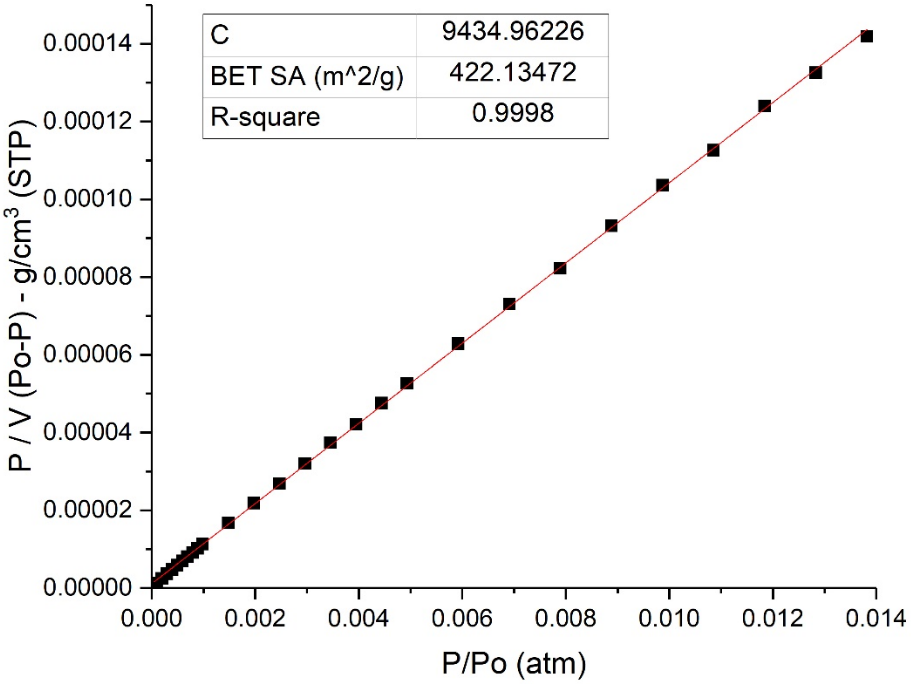

Supplementary Figure 3. Top: Simulated N_2 isotherm. Bottom: Plot of the linear region for the BET equation.

Electronic structure. The geometry optimization of **GTUB5** was performed using density functional theory (DFT) and the conjugate gradient method[13] within the Quickstep-CP2K program[14,15], starting from the experimental crystal structure and with the lattice vectors set to their experimental values. Since **GTUB5** is a bulk material, periodic boundary conditions were applied to a reoriented 1x1x1 cell (a=25.452 Å, b=22.863 Å, c=7.1798 Å, α=γ= 90.0°, β = 102.325°). The Perdew-Burke-Ernzerhof (PBE)[7] generalized gradient approximation (GGA) functional was used in conjunction with the Grimme D3 dispersion correction[16] and BJ damping[17]. The Gaussian and plane waves method[15,18] was used, with the valence orbitals expanded in terms of molecularly optimized Gaussian basis sets of double-ζ plus polarization (MOLOPT-DZVP)[19] quality and the core electrons represented by norm-conserving Goedecker-Teter-Hutter pseudopotentials[20,21]. Γ-point sampling was used and the plane-wave cutoff in reciprocal space was set to 550 Ry, with a Gaussian mapping of 60 Ry over five multi-grids. The self-consistent field was converged to 10^{-6} Ry with the FULL_ALL preconditioner using the orbital transformation method with a HOMO-LUMO gap of 1.67 eV. Single point calculations were performed using CP2K to obtain the HOMO-LUMO iso-surface plots (Figures 2 and 3 in the main text), orbital populations (Table 1 of main text), and HOMO-LUMO gap. A second single point calculation was performed using the Slater-Type Orbital (STO) software ADF-BAND 2018.104[22,23] to obtain the projected density of states (pDOS) (Figure 4 of main text), band structure (Supplementary Figure 8), and band gap. The periodic ADF-BAND calculations were performed using an all-electron double-ζ plus polarization (DZP) basis set, PBE-D3-BJ, and Γ-point sampling for the 1x1x1 unit cell, with good numerical quality. The HOMO-LUMO gaps obtained from CP2K and ADF-BAND were both 1.65 eV (thus, the HOMO-LUMO iso-surfaces and orbital populations obtained from CP2K are expected to be the same as those from ADF-BAND).

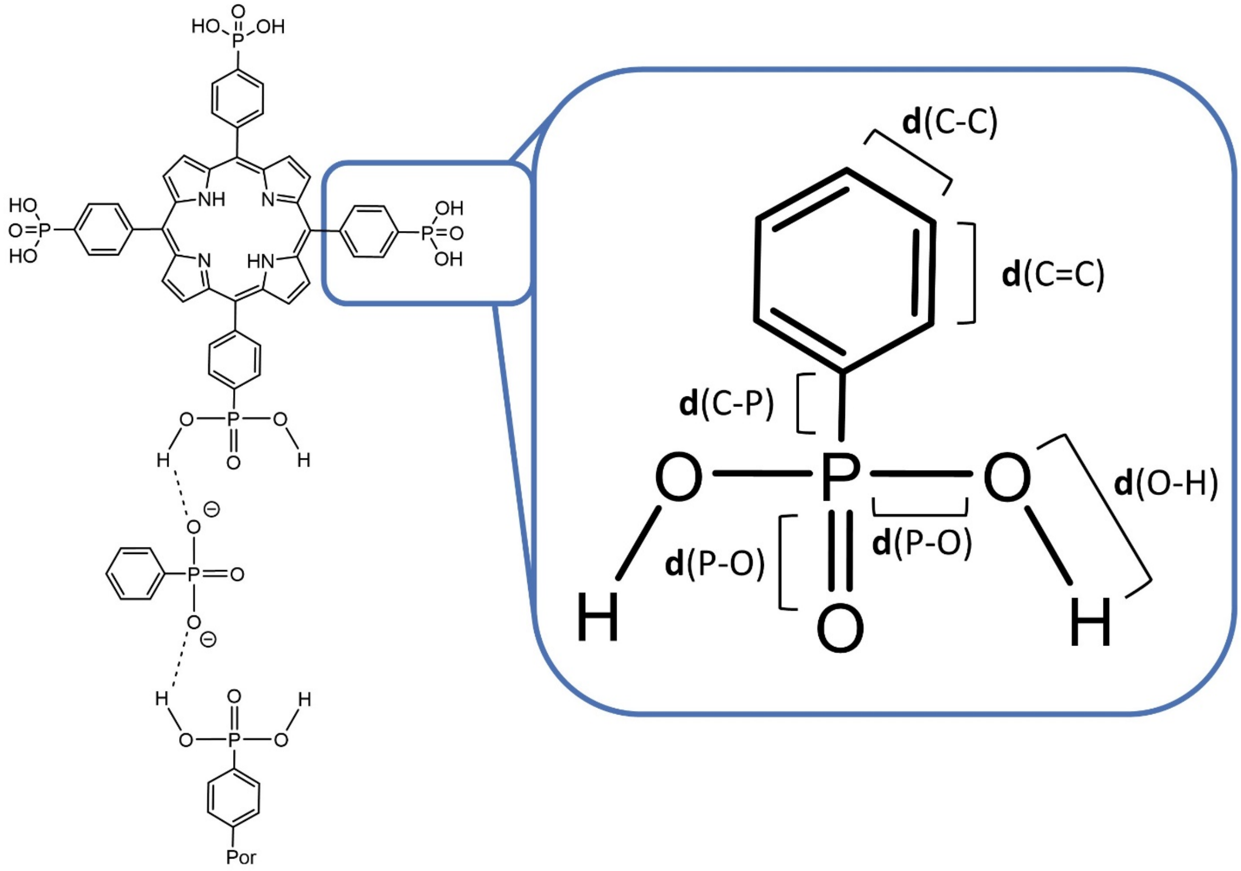

Supplementary Figure 4. Simplified chemical structure of the **GTUB5** building block, highlighting one of the phenyl-phosphonic acid groups. The bond distance labeling is used in Supplementary Table 3.

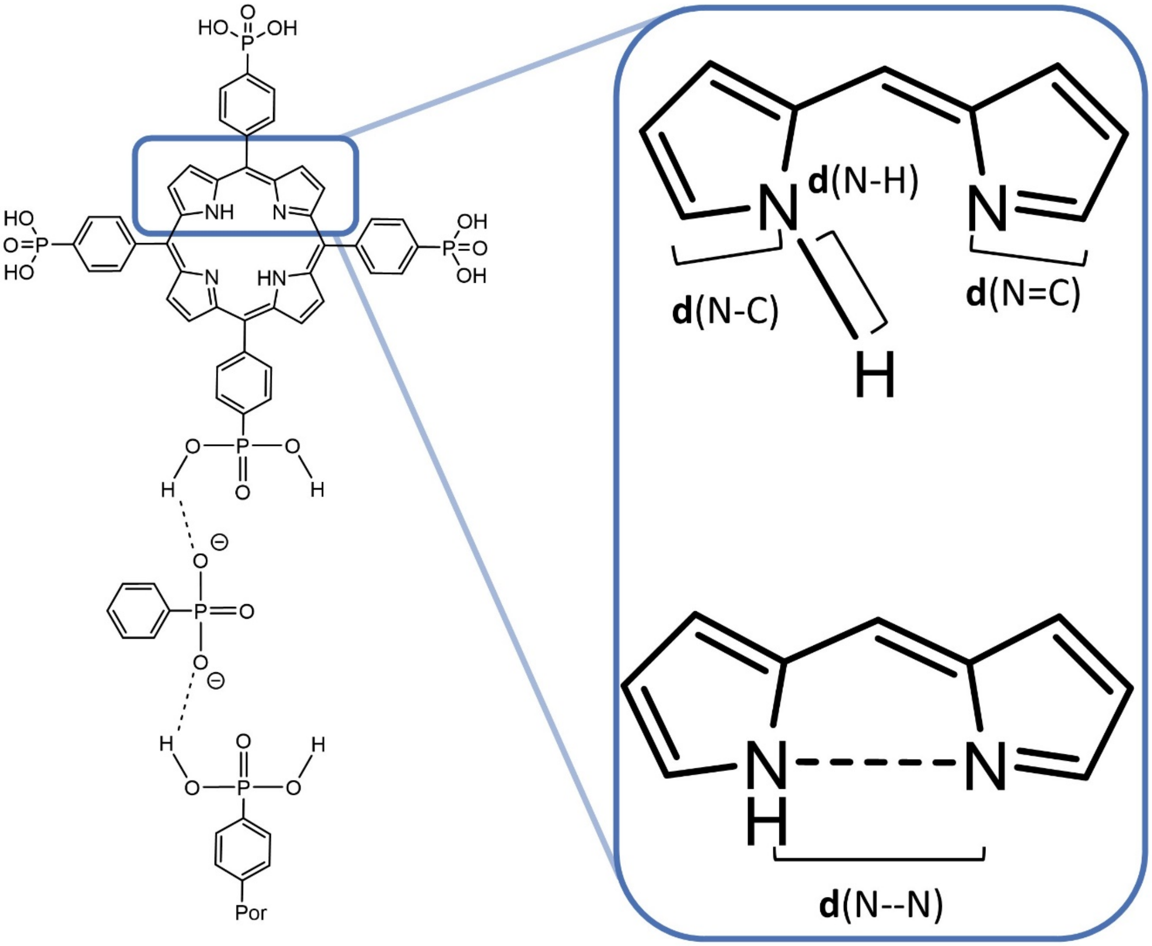

Supplementary Figure 5. Simplified chemical structure of the **GTUB5** building block, highlighting the dipyrromethene portion of the porphyrin group. The bond distance labeling is used in Supplementary Table 3.

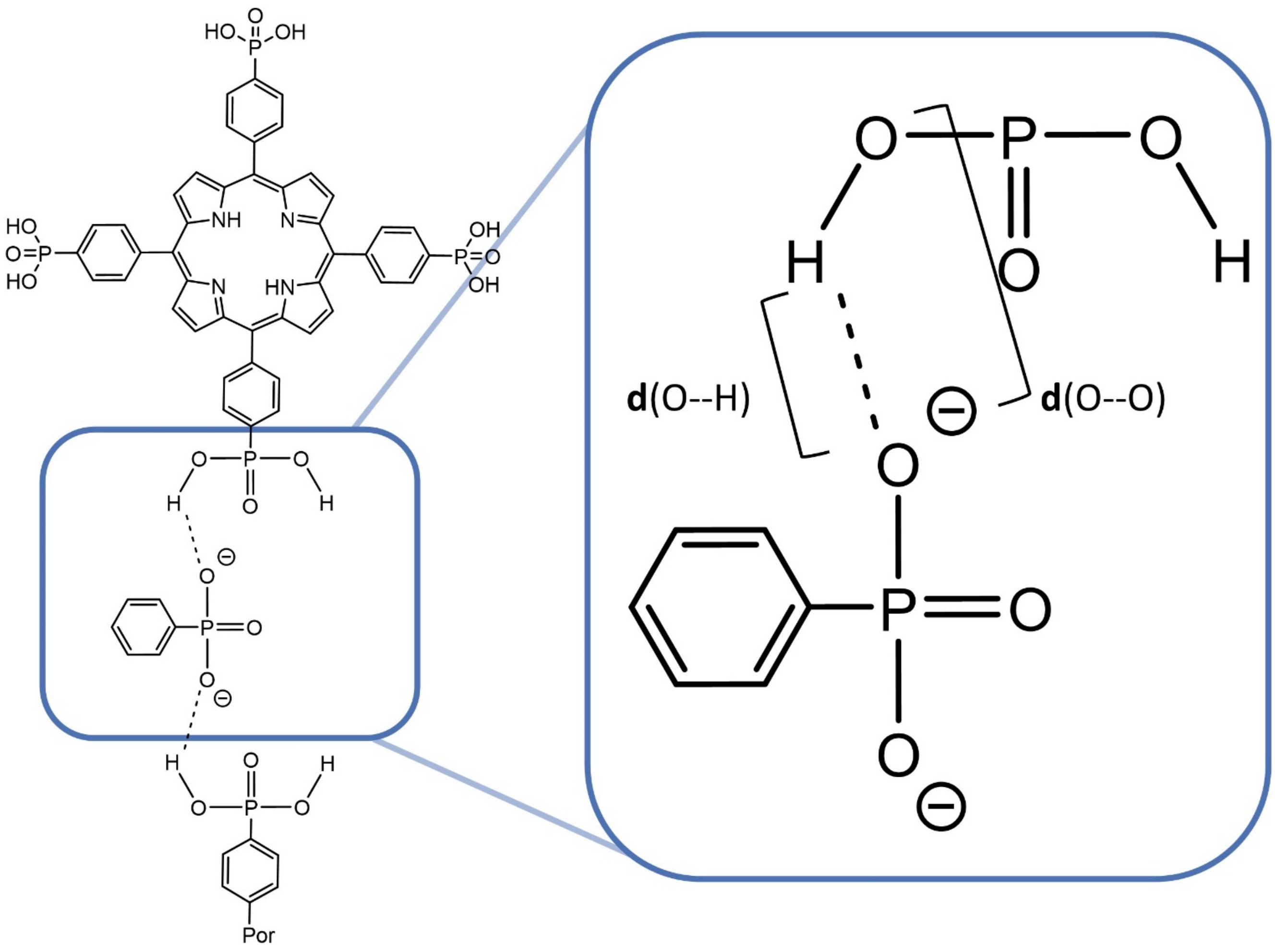

Supplementary Figure 6. Simplified chemical structure of the GTUB5 building block, highlighting the hydrogen bonding between neighbouring phenyl-phosphonic acid groups. The bond distance labeling is used in Supplementary Table 3.

Supplementary Table 3. Comparison of experimental and calculated average inter-atomic distances (in Å). Standard deviations in distances are given in brackets. The calculated structure was obtained from a geometry optimization of the experimental crystal structure at the PBE-D3-BJ DZVP-550 Ry level of theory.

Atom pair	Experimental	Calculated
Supplementary Figure 4		
C-P	1.78<br>(0.018)	1.80<br>(0.008)
P-O	1.53<br>(0.003)	1.58<br>(0.013)
O-H	0.83<br>(0.013)	1.16<br>(0.096)
C-C	1.49<br>(0.000)	1.46<br>(0.023)
C=C	1.39<br>(0.025)	1.40<br>(0.01)
Supplementary Figure 5		
N-C	1.78<br>(0.018)	1.80<br>(0.008)
N=C	1.53<br>(0.003)	1.58<br>(0.013)
N-H	0.88	1.05

	(0.012)	(0.015)
N--N	2.92<br><br>(0.000)	2.93<br><br>(0.000)
Supplementary Figure 6		
O-O	2.47<br><br>(0.018)	2.43<br><br>(0.000)
O--H	1.88<br><br>(0.164)	1.75<br><br>(0.005)

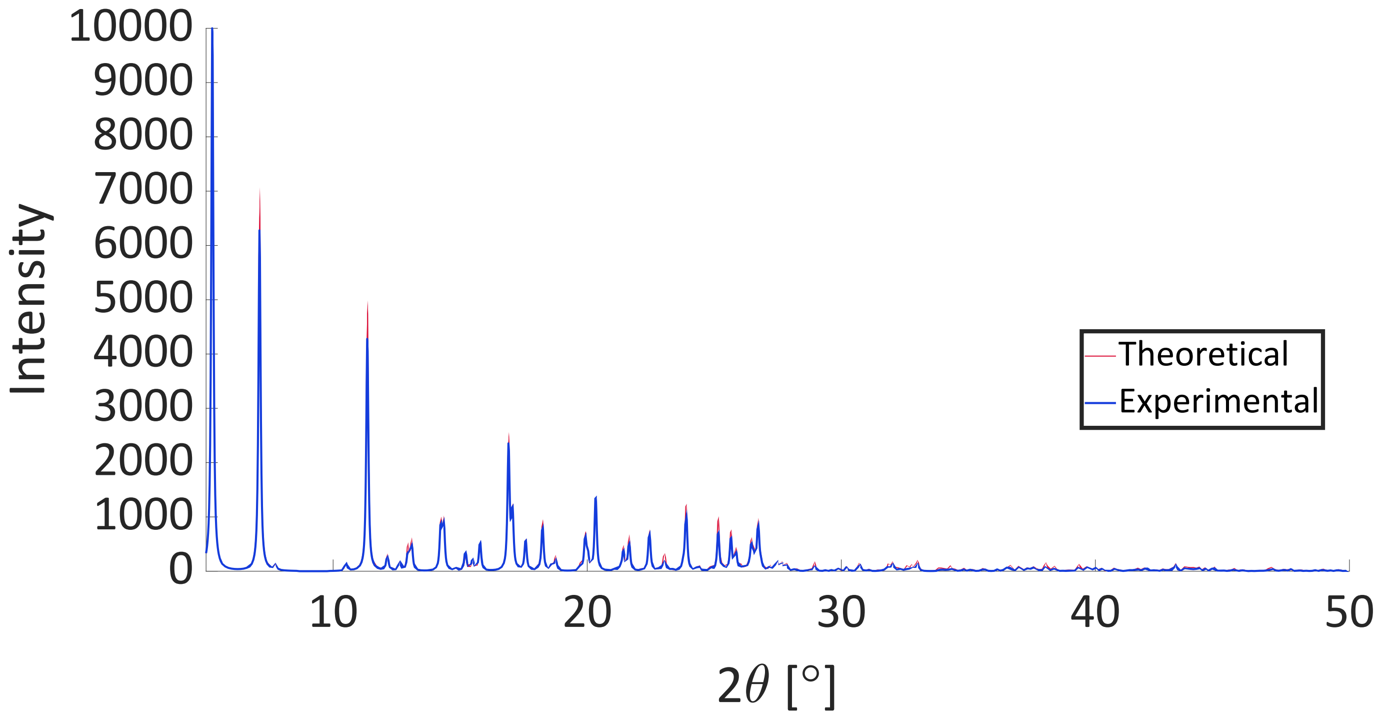

Supplementary Figure 7. Comparison between experimental and theoretical X-ray diffraction patterns. The theoretical result was generated using a full width-half max of 0.1 $2\theta°$ within the Mercury software package[24].

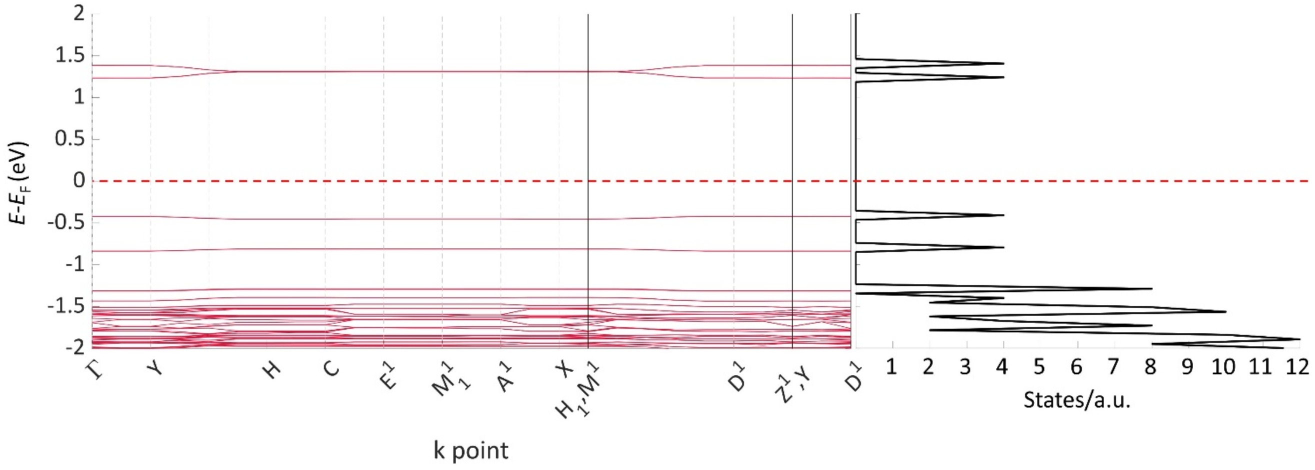

Supplementary Figure 8. (Left) Band structure of GTUB5. **(Right)** The corresponding total density of states (DOS). Both were obtained using ADF-BAND version 2018.104[22,23].

X-ray data collection and structure refinement

Data for **GTUB-5** was obtained with a Bruker APEX II QUAZAR three-circle diffractometer. Indexing was performed using APEX2[25]. Data integration and reduction were carried out with SAINT[26]. Absorption correction was performed by the multi-scan method implemented in SADABS[27]. The structure was solved using SHELXT[28] and then refined by full-matrix least-squares refinements on F^2 using the SHELXL[29] in the Olex2 software package[30]. The positions of all H-atoms bonded to carbon, nitrogen, and oxygen atoms were geometrically optimized with the following HFIX instructions in SHELXL: HFIX 23 for the $–CH_2–$ moieties, HFIX 137 for the $–CH_3$, HFIX 43 for the CH and NH groups of the aromatic rings and porphyrin cores, and HFIX 147 for the –P–OH groups (H1a) of the phosphonic acid moieties. Another O-bound H atom (H3) was located from a difference Fourier-map. Finally, their displacement parameters were set to isotropic thermal displacements parameters (U_{iso}(H) = 1.2×U_{eq} for CH, NH and CH_2 groups or (U_{iso}(H) = 1.5×U_{eq} (-OH and CH_3 groups). In the chemical formula [(H_8-TPPA)$(PPA)_2(DMA)_4$] of **GTUB-5**, the **H_8-TPPA** building block is not deprotonated, while the protons

of the phenylphosphonic acid (PPA) groups are acquired by the DMF solvent in the pores to form four dimethylammonium cations (DMA – $[NH_2(CH_3)_2]^+$) to balance the charge. SQUEEZE was used to remove electron density caused by seriously disordered solvent molecules in **GTUB-5**. Along the *c*-axis, the 3D supramolecular network of **GTUB-5** produced a one-dimensional distinctive void space with a total potential solvent area occupying 19.2% (785 Å^3) of the unit cell volume (4081.7 Å^3) obtained using the PLATON software package[31]. Analysis of solvent accessible voids in the structure was performed using the CALC SOLV within PLATON with a probe radius of 1.20 Å and grid spacing of 0.2 Å. Van der Waals (or ion) radii used in the analysis are 1.70 Å for C, 1.20 Å for H, 1.55 Å for N, 1.52 Å for O, and 1.80 Å for P. Also, in this crystal structure, the rotationally disordered phosphonate part ($-PO_3$) in phenylphosphonic acid (PPA) was refined as 0.77:0.23. Crystallographic data and refinement details of the data collection for **GTUB-5** are given in Supplementary Table 4. Crystal structure validations and geometrical calculations were performed using PLATON[31]. The Mercury software package[24] was used for visualization of the cif files. Additional crystallographic data with CCDC reference numbers (1963794 for **GTUB-5**) was deposited into the Cambridge Crystallographic Data Center at www.ccdc.cam.ac.uk/deposit.

Supplementary Table 4. X-ray crystallographic data and refinement parameters for **GTUB-5**.

CCDC	1963794
Empirical formula	$C_{64}H_{76}N_8O_{18}P_6$
Formula weight (g mol^{-1})	1431.14
Temperature (K)	296
Radiation, Wavelength (Å)	MoK_α (λ = 0.71073)
Crystal system	Monoclinic

Space group	C2/m
a (Å)	25.452(2)
b (Å)	22.863(2)
c (Å)	7.1798(6)
α (°)	90
β (°)	102.325(6)
γ (°)	90
Crystal size (mm^3)	0.43 × 0.14 × 0.12
Volume (Å^3)	4081.7(6)
Z	2
ρ_{calcd} (g cm^{-3})	1.164
μ (mm^{-1})	0.195
F (000)	1500
2θ range for data collection (°)	5.96 to 50.04
h/*k*/*l*	$-30 \le h \le 30$, $-27 \le k \le 27$, $-8 \le l \le 8$
Reflections collected	21748
Independent reflections	3692 [R_{int} = 0.0572, R_{sigma} = 0.0449]
Data/restraints/parameters	3692/33/247
Goodness-of-fit on F^2 (S)	1.036
Final R indices [$I > 2\sigma(I)$]	R_1 = 0.0769, wR_2 = 0.2197
R indices (all data)	R_1 = 0.1108, wR_2 = 0.2485
Largest diff. peak/hole / e Å^{-3}	0.47/-0.38

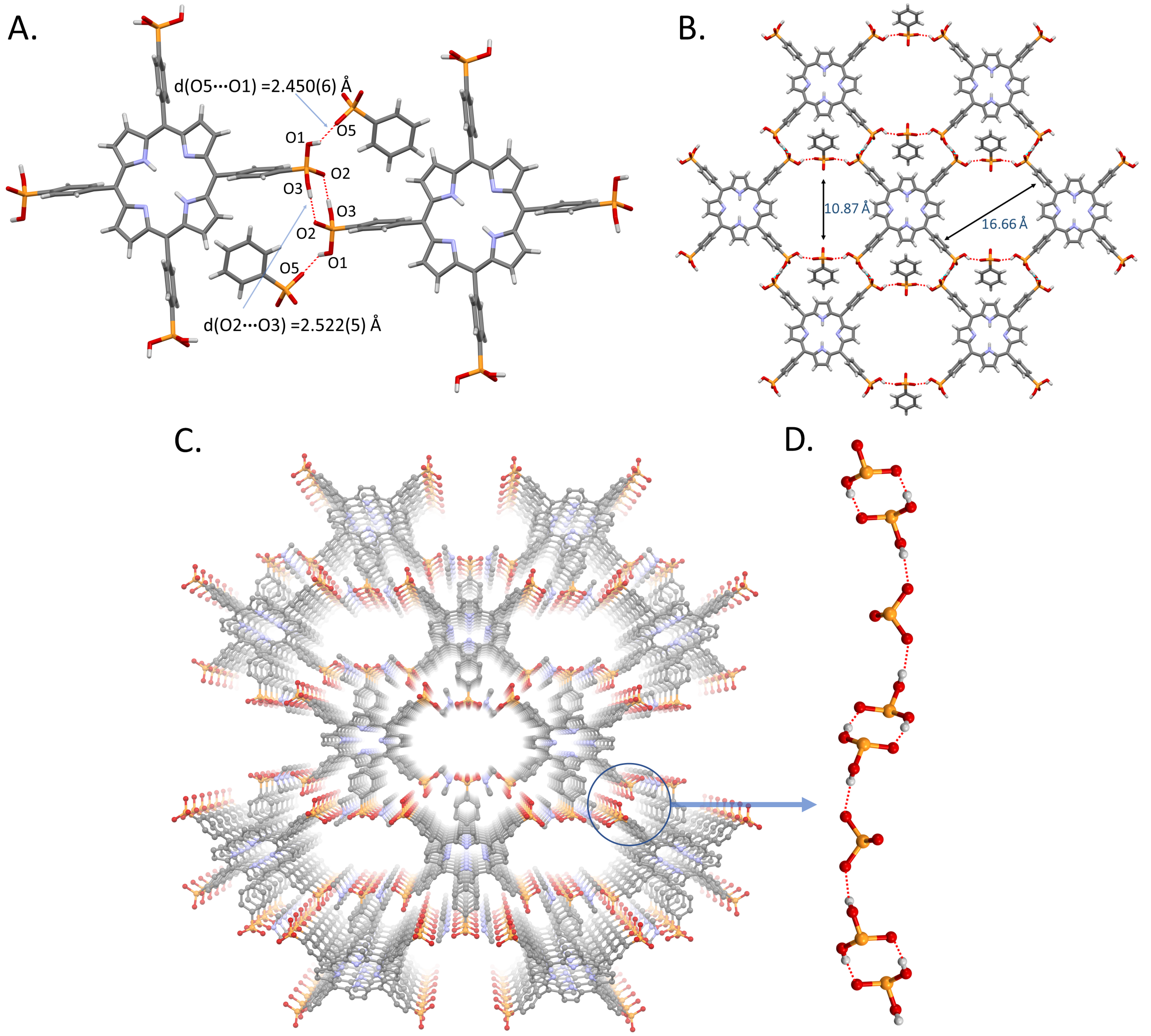

Supplementary Figure 9. (A) View of hydrogen bonding interactions between PPA and H_8-TPPA linkers. (B) Two-dimensional hydrogen-bonded layers constructed from hydrogen bonding interactions between PPA and H_8-TPPA linkers. (C) Three-dimensional hydrogen-bonded framework of **GTUB-5** along the *c*-axis. (D) View of one-dimensional infinite hydrogen-bonded chain.

Supplementary Table 5. Hydrogen bond parameters (in Å and °) for **GTUB-5**.

**D-H···A**	***d*(D-H)**	***d*(H···A)**	***d*(D-H···A)**	**∠ D-H···A**
O1-H1A···O5^{i}	0.82	1.74	2.450(6)	144.05
O3-H3···O2^{ii}	0.85	1.68	2.522(5)	171.88
N3-H3B···O4^{iii}	0.89	2.06	2.945(13)	170.41
N3-H3C···O5^{iv}	0.89	2.08	2.956(12)	167.22

Symmetry codes: (i) 3/2-x,3/2-y,1-z; (ii) 3/2-x,3/2-y,2-z; (iii) 3/2-x,-1/2+y,1-z; (iv) 3/2-x,3/2-y,-z.

Thermogravimetric analysis (TGA)

TGA on **GTUB-5** was performed using a Mettler-Toledo TGA/DSC STARe System at a heating rate of 10 K min^{-1} under an atmosphere of dry argon over a range from 50 to 700 °C.

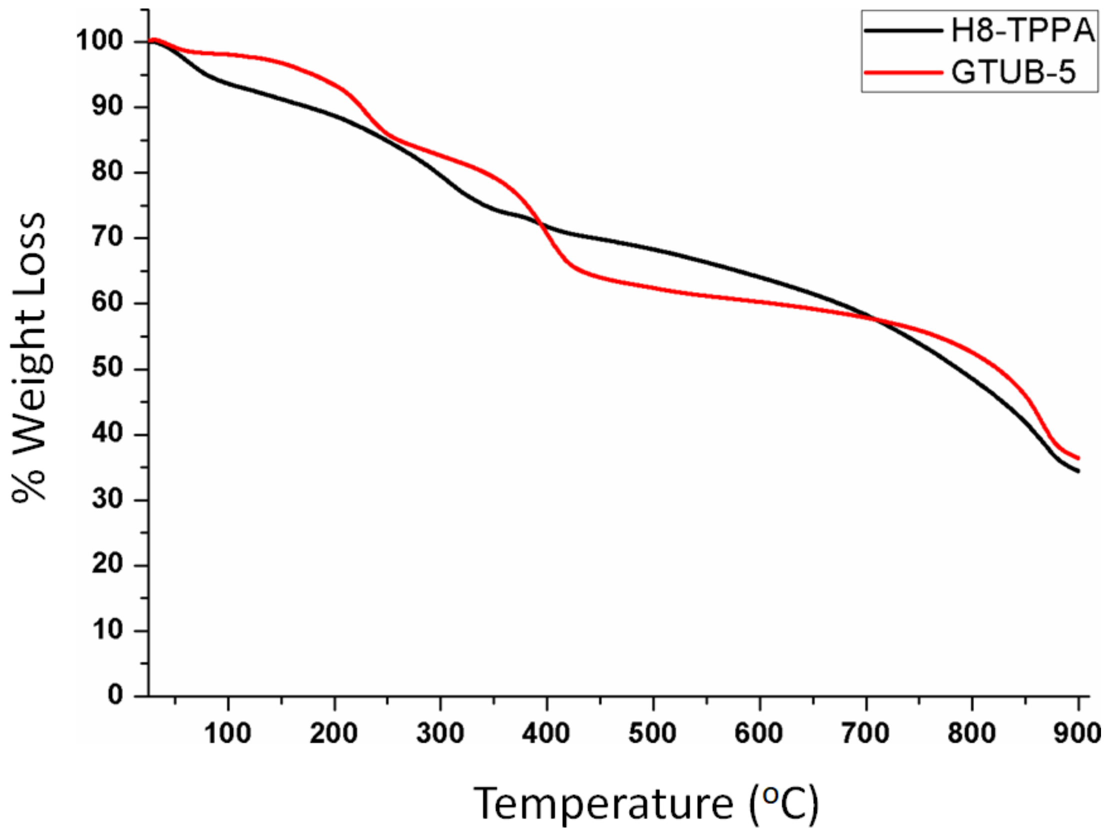

Supplementary Figure 10. Thermogravimetric curves of **H_8TPPA** and **GTUB-5** between room temperature and 700 °C with a heating rate of 10 °C min^{-1}.

FT-IR spectroscopy

IR spectra of **H_8TPPA** and **GTUB-5** were recorded between 4000 and 550 cm^{-1} using a Perkin Elmer Spectrum 100 FT-IR spectrometer with an attenuated total reflection (ATR) accessory featuring a zinc selenide (ZnSe) crystal.

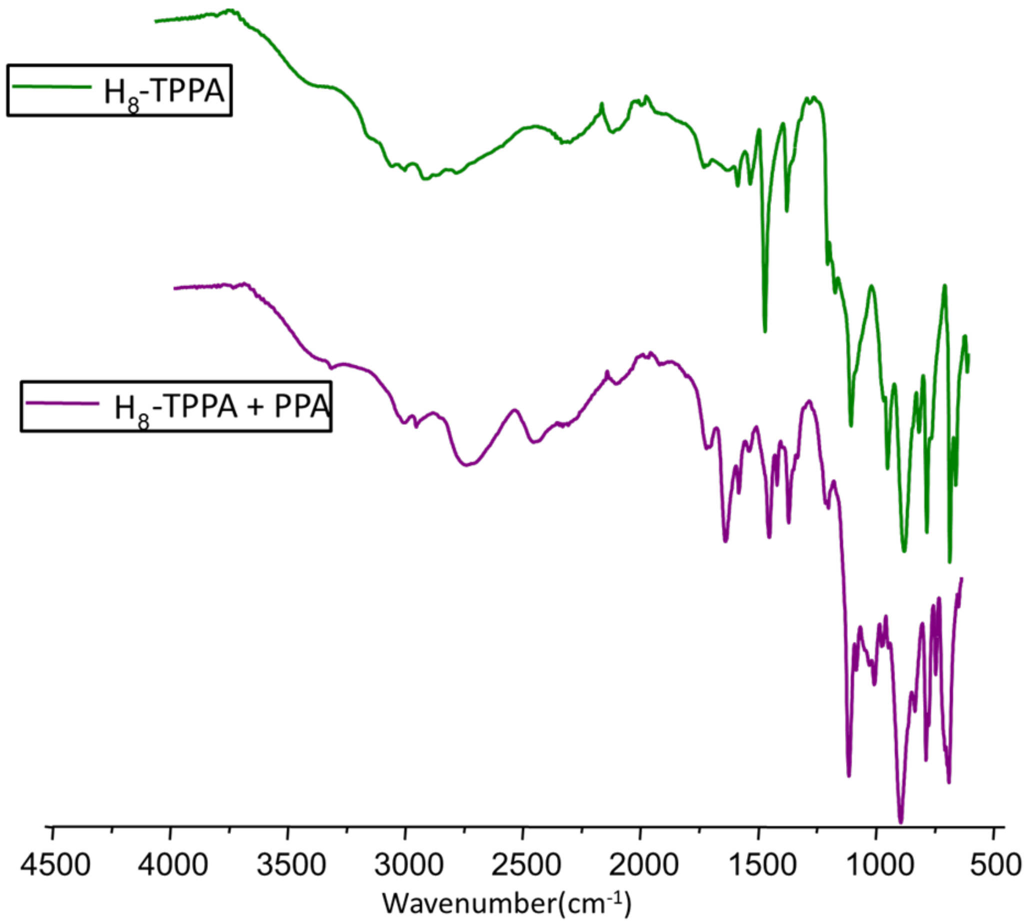

Supplementary Figure 11. FT-IR spectra of **H_8-TPPA** and **H_8TPPA-PPA** (**GTUB-5**).

UV-Vis spectroscopy

The solid-state diffuse reflectance ultraviolet–visible (UV-Vis) spectrum of **GTUB-5** crystals was collected on a Varian Cary 300 UV-Vis Spectrophotometer and the corresponding solution spectrum was collected using a Varian Eclipse spectrofluorometer with 1-cm path length cuvettes at room temperature in DMSO.

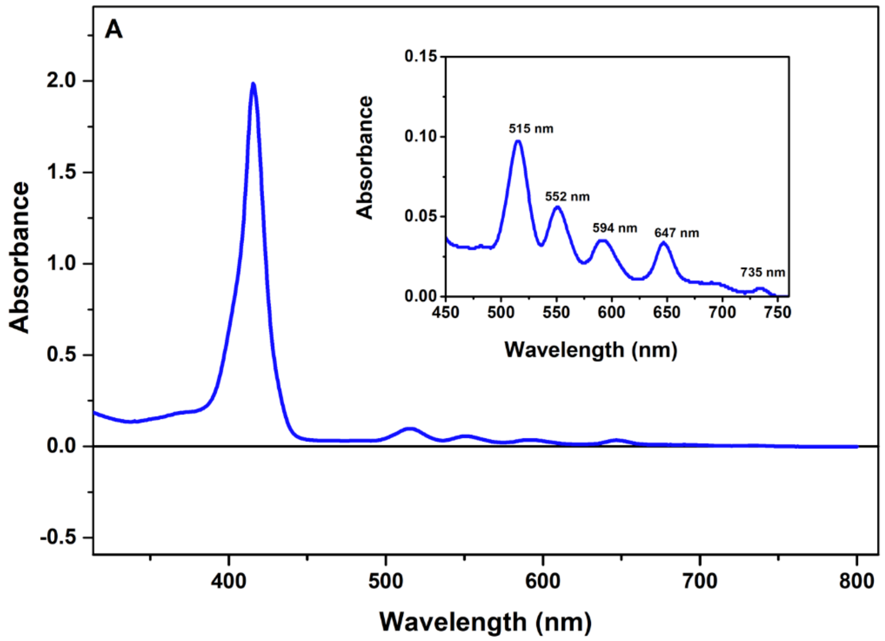

Supplementary Figure 12. UV-Vis spectrum of **GTUB-5** in DMSO.

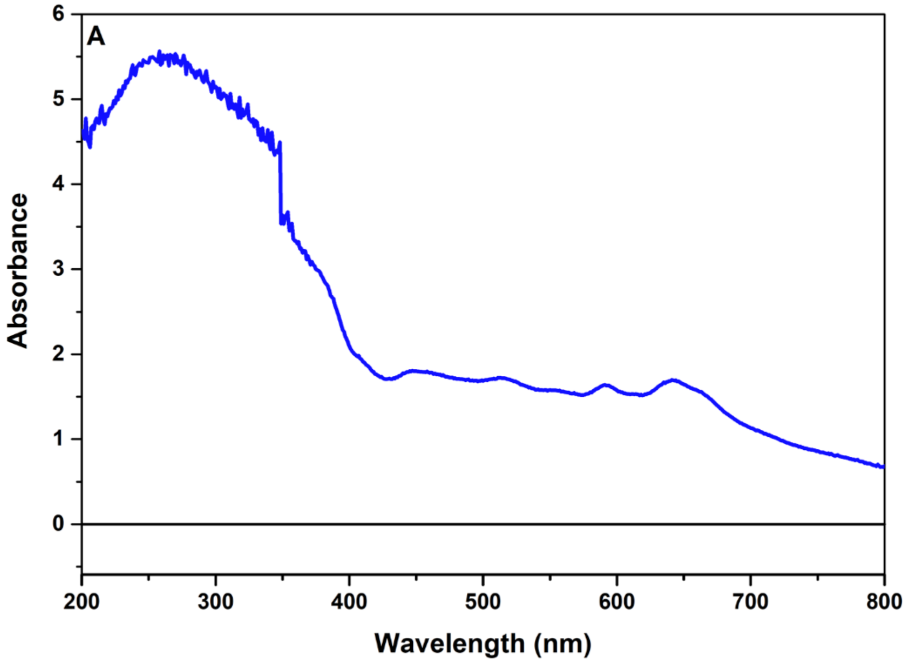

Supplementary Figure 13. Solid-state UV-Vis spectrum of **GTUB-5**.

Cyclic voltammetry

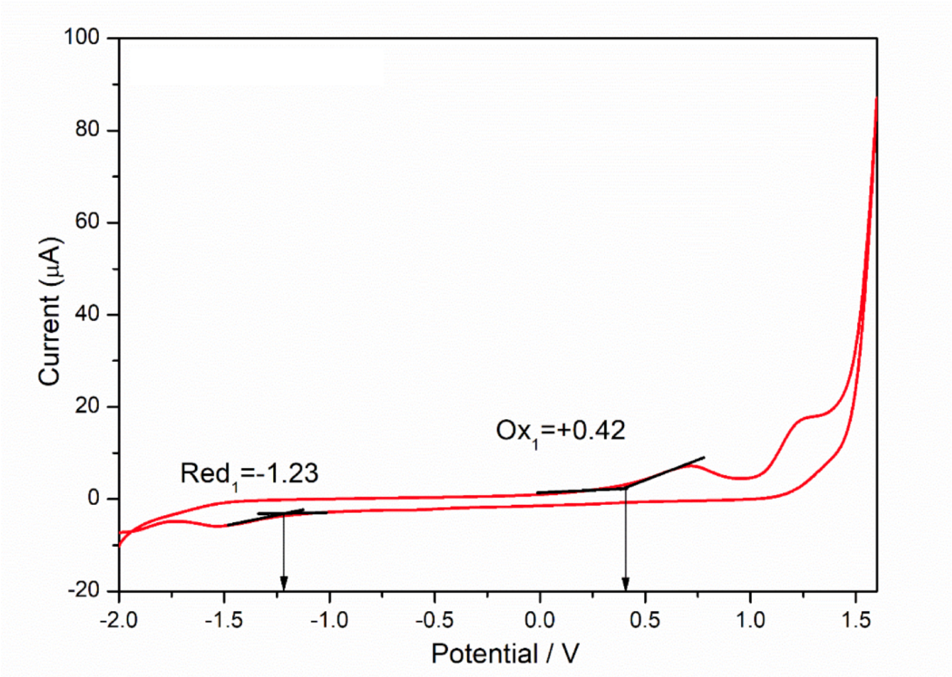

Supplementary Figure 14. Cyclic voltammetry of **GTUB-5**.

The HOMO-LUMO gap of **GTUB-5** was extracted using cyclic voltammetry (See Supplementary Figure 14)[32]. From the measurement, the first oxidation and reduction potentials of **GTUB-5** in DMSO were determined to be 0.42 V and -1.23 V, which give rise to a HOMO-LUMO gap of 1.65 eV.

Proton conductivity measurement

The proton conductivity of **GTUB-5** was determined by electrochemical impedance spectroscopy. A Zahner Zennium electrochemical workstation was used with an oscillation voltage of 10 mV over a frequency from 1 to 10^6 Hz. The needles were compressed between two glassy carbon electrodes by a torque of 30 cNm to obtain pellets of 82 mm in diameter and ca. 0.114 mm thickness. The stack was placed in a PTFE sample holder. The sample holder was placed in a stainless-steel chamber with an attached water reservoir. The relative humidity (%rh) was determined by the Clausius-Clapeyron relation and controlled by heating the cell and water reservoir. The sample is held overnight at the desired %rh and temperature before measuring each data point. To ensure reproducibility, each data point was measured three times.

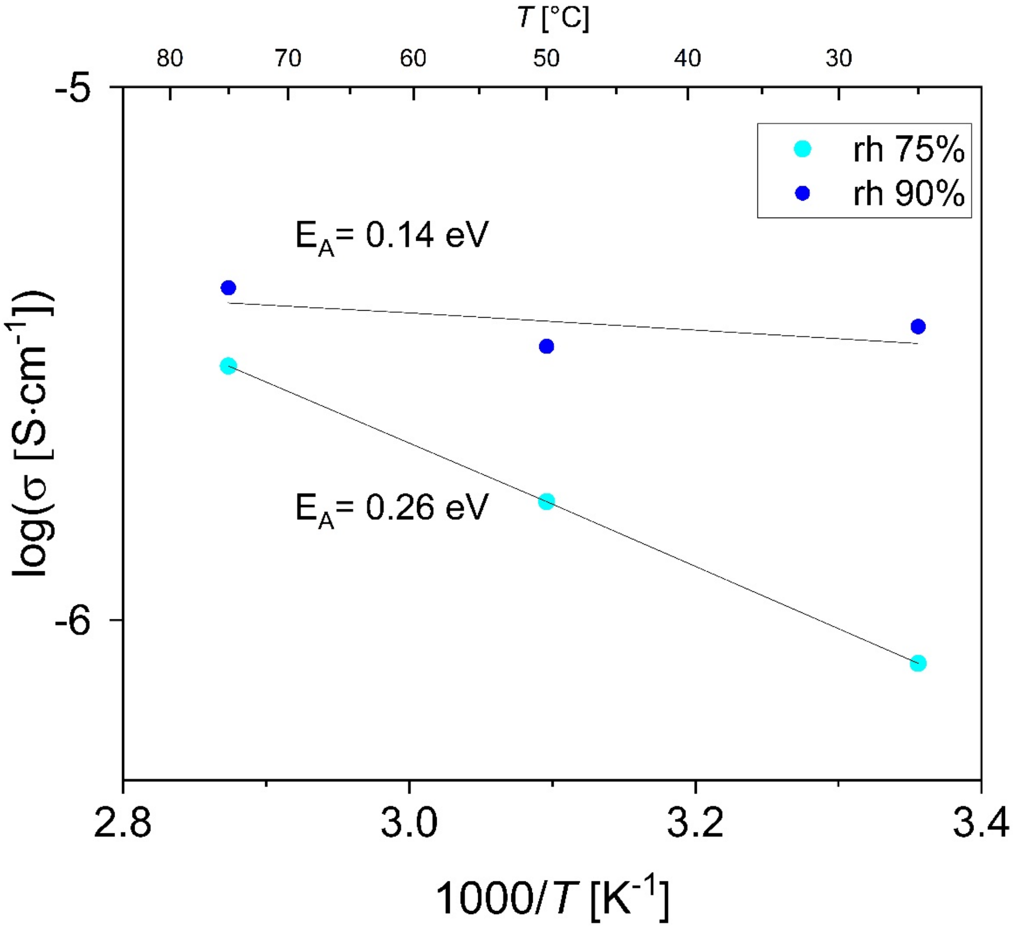

Supplementary Figure 15. Proton conductivity of **GTUB-5**.

Supplementary Figure 16 shows a Bode plot from which the proton conductivity at 75 °C 90 %rh was determined to be $\sigma = 4.20 \times 10^{-6}$ S cm^{-1}.

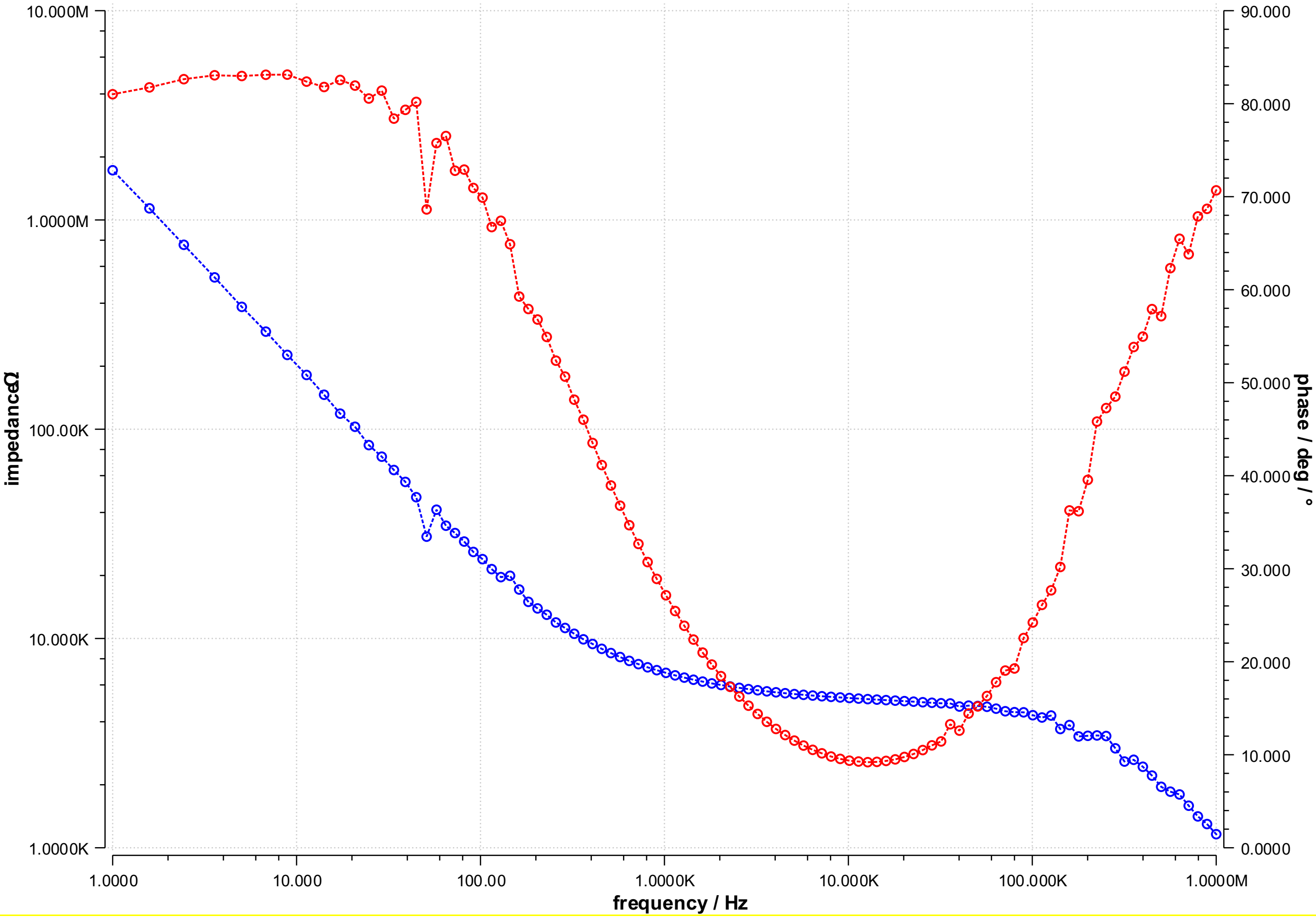

Supplementary Figure 16. Bode plot of **GTUB-5** at 75 °C and 90 %rh.

Powder x-ray diffraction

PXRD patterns of the **GTUB-5** sample were measured on a PANalytical X'pert PRO theta-theta x-ray diffractometer (Mavern Panalytical B.B., Almelo, Netherlands) operation at 40 kV and 40 mA, before and after the proton conductivity experiment. The sample was placed on a silicon zero background sample holder. Measurements were performed in the range of 3–50 2*θ*° with a step size of 0.026 2*θ*° and a counting time of 246.840 s. The results of the measurements were processed with the software Highscore plus version 4.8.

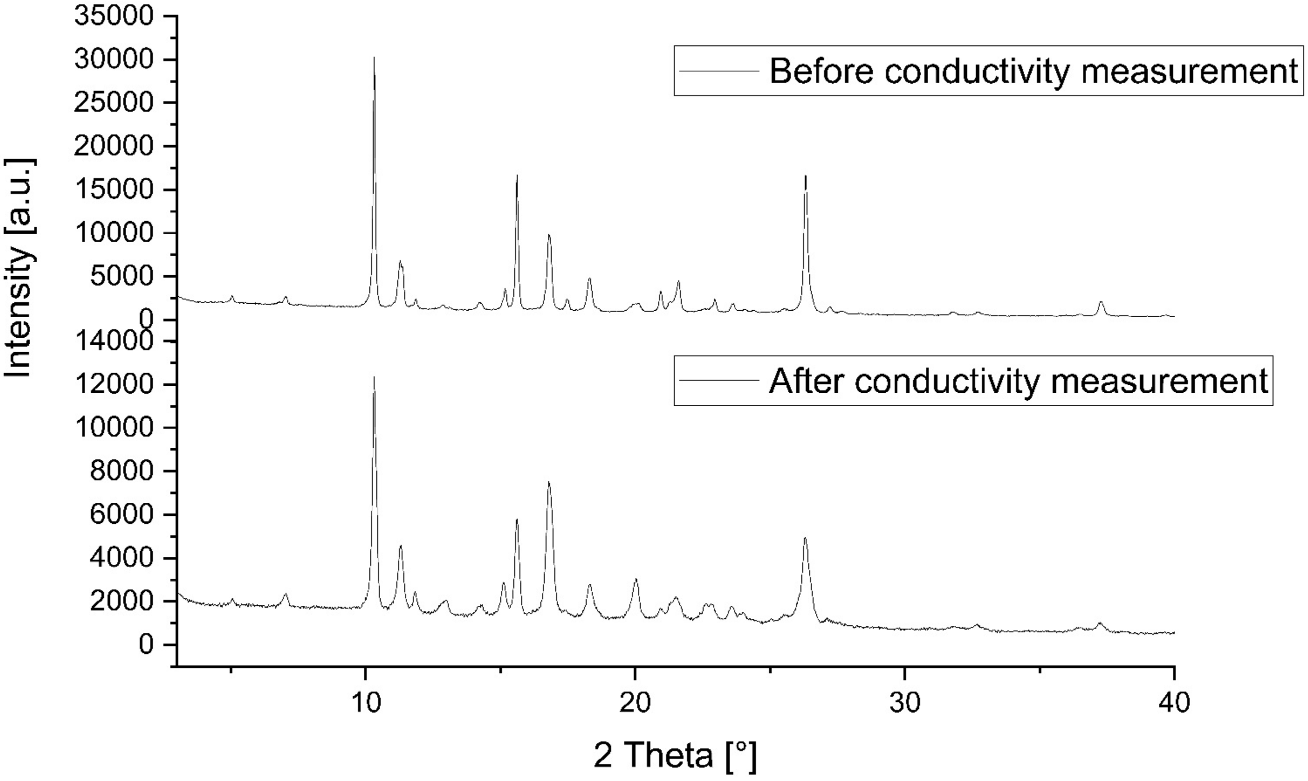

Supplementary Figure 17. Powder x-ray diffraction of **GTUB-5** before and after the proton conductivity experiment.

Supplementary References

1 Maares, M. et al. Alkali Phosphonate Metal-Organic Frameworks. *Chem. Eur. J.* **25**, 11214-11217 (2019).

2 Dubbeldam, D., Calero, S., Ellis, D. E. & Snurr R. Q. RASPA: molecular simulation software for adsorption and diffusion in flexible nanoporous materials. *Mol. Sim.* **42**, 81-101 (2016).

3 Mayo, S. L., Olafson, B. D., Goddard, W. A. DREIDING: a generic force field for molecular simulations. *J. Phys. Chem*. **94**, 8897-8909 (1990).

4 Campaná, C., Mussard, B., Woo, T. K. Electrostatic Potential Derived Atomic Charges for Periodic Systems Using a Modified Error Functional. *J. Chem. Theo. Comp*. **5**, 2866-2878 (2009).

5 Clark, S. J. et al. First principles methods using CASTEP. *Z. Krist*. **220**, 567-570 (2005).

6 Perdew, J., Burke, K., Ernzerhof, M. Generalized Gradient Approximation Made Simple. *Phys. Rev. Lett.* **77**, 3865-3868 (1996).

7 Vanderbilt, D. Soft self-consistent pseudopotentials in a generalized eigenvalue formalism. *Phys. Rev. B, Condens. Matter*, **41**, 7892–7895 (1990).

8 Talu, O., Myers, A. L. Molecular simulation of adsorption: Gibbs dividing surface and comparison with experiment. *AIChE. J.* **47**, 1160-1168 (2001).

9 Hirschfelder, J. O., Curtiss, C. F., Bird, R. B. *Molecular Theory of Gases and Liquids*, (Wiley, New York, USA, 1954, p. 1114).

10 Gelb, L. D., Gubbins, K. E. Pore Size Distributions in Porous Glasses: A Computer Simulation Study. *Langmuir* **15**, 305-308 (1999).

11 Potoff, J. J., Siepmann, J. I. Vapor-liquid equilibria of mixtures containing alkanes, carbon dioxide, and nitrogen. *AIChE J.* **47**, 1676-1682 (2001).

12 Walton, K. S., Snurr, R. Q. Applicability of the BET Method for Determining Surface Areas of Microporous Metal-Organic Frameworks. *J. Am. Chem. Soc*. **129**, 8552-8556 (2007).

13 Hestenes, M. R., Stiefel, E., Methods of conjugate gradients for solving linear systems. *J. Res. Natl. Bur. Stand.* **49**, 409-436 (1952).

14 VandeVondele, J., Krack, M., Mohamed, F., Parrinello, M., Chassaing, T., & Hutter, J. Quickstep: Fast and accurate density functional calculations using a mixed Gaussian and plane waves approach. *Comput. Phys. Commun.* **167**, 103–128 (2005).

15 Hutter, J., Iannuzzi, M., Schiffmann, F., & Vandevondele, J. CP2K: Atomistic simulations of condensed matter systems. *WIRES Comput. Mol. Sci.* **4**, 15–25 (2014).

16 Grimme, S., Antony, J., Ehrlich, S., & Krieg, H. A consistent and accurate ab initio parametrization of density functional dispersion correction (DFT-D) for the 94 elements H-Pu. *J. Chem. Phys.* **132**, 154104-1-154104–19 (2010).

17 Grimme, S., Ehrlich, S., & Goerigk, L. Effect of the damping function in dispersion corrected density functional theory. *J. Comput. Chem.* **32**, 1456–1465 (2011)

18 Lippert, G., Hutter, J., & Parrinello, M. A hybrid Gaussian and plane wave density functional scheme. *Mol. Phys.* **92**, 477–488 (1997)

19 VandeVondele, J., & Hutter, J. Gaussian basis sets for accurate calculations on molecular systems in gas and condensed phases. *J. Chem. Phys.* **127**, 114105-1-114105-9 (2007).

20 Goedecker, S., & Teter, M. Separable dual-space Gaussian pseudopotentials. *Phys. Rev. B, Condens. Matter*, **54**, 1703–1710 (1996).

21 Hartwigsen, C., Goedecker, S., Hutter, J., Relativistic separable dual-space gaussian pseudopotentials from H to Rn. *Phys. Rev. B, Condens. Matter* **58**, 3641–3662 (1998).

22 Philipsen, P. H. T. et al. 2018. BAND 2018.104, SCM, Theoretical Chemistry, Vrije Universiteit, Amsterdam, The Netherlands, http://www.scm.com

23 te Velde, G. et al. Chemistry with ADF. *J. Comput. Chem.* **22**, 931–967 (2001).

24 Macrae, C. F et al. Mercury: Visualization and analysis of crystal structures. *J. Appl. Crystallogr.* **39**, 453–457 (2006).

25 APEX2, version 2014.11-0, Bruker (2014), Bruker AXS Inc., Madison, WI.

26 SAINT, version 8.34A, Bruker (2013), Bruker AXS Inc., Madison, WI.

27 SADABS, version2014/5, Bruker (2014), Bruker AXS Inc., Madison, WI.

28 Sheldrick, G. M. SHELXT – Integrated space-group and crystal-structure determination. *Acta Cryst. A* **A71**, 3-8 (2015).

29 Sheldrick, G. M. Crystal structure refinement with SHELXL. *Acta Cryst. C* **C71**, 3-8 (2015)

30 Dolomanov, O. V., Bourhis, L. J., Gildea, R. J., Howard, J. A. K. & Puschmann H., *J. Appl. Crystallogr.* **42** 336-338 (2009).

31 Spek, A. L. Structure validation in chemical crystallography. *Acta Cryst. D* **D65**, 148-155 (2009).

32 Kadish, K. M., Caemelbecke, E. V. Electrochemistry of porphyrins and related macrocycles. *J. Solid State Electr.* **7**, 254-258 (2003).
